# Use of Baseline ^*18*^F-FDG PET/CT to Identify Initial Sub-Volumes Associated With Local Failure After Concomitant Chemoradiotherapy in Locally Advanced Cervical Cancer

**DOI:** 10.3389/fonc.2020.00678

**Published:** 2020-05-07

**Authors:** François Lucia, Omar Miranda, Ronan Abgral, Vincent Bourbonne, Gurvan Dissaux, Olivier Pradier, Mathieu Hatt, Ulrike Schick

**Affiliations:** ^1^Radiation Oncology Department, University Hospital, Brest, France; ^2^LaTIM, INSERM, UMR 1101, Univ Brest, Brest, France; ^3^Nuclear Medicine Department, University Hospital, Brest, France

**Keywords:** PET/CT, cervical cancer, chemoradiotherapy, hotspots, personalized targeted treatment

## Abstract

**Introduction:** Locally advanced cervical cancer (CC) patients treated by chemoradiotherapy (CRT) have a significant local recurrence rate. The objective of this work was to assess the overlap between the initial high-uptake sub-volume (V1) on baseline ^18^F-fluorodeoxyglucose (FDG) positron emission tomography/computed tomography (PET/CT) scans and the metabolic relapse (V2) sites after CRT in locally advanced CC.

**Methods:** PET/CT performed before treatment and at relapse in 21 patients diagnosed with LACC and treated with CRT were retrospectively analyzed. CT images at the time of recurrence were registered to baseline CT using the 3D Slicer TM Expert Automated Registration module. The corresponding PET images were then registered using the corresponding transform. The fuzzy locally adaptive Bayesian (FLAB) algorithm was implemented using 3 classes (one for the background and the other two for tumor) in PET1 to simultaneously define an overall tumor volume and the sub-volume V1. In PET2, FLAB was implemented using 2 classes (one for background, one for tumor), in order to define V2. Four indices were used to determine the overlap between V1 and V2 (Dice coefficients, overlap fraction, X = (V1nV2)/V1 and Y = (V1nV2)/V2).

**Results:** The mean (±standard deviation) follow-up was 26 ± 11 months. The measured overlaps between V1 and V2 were moderate to good according to the four metrics, with 0.62–0.81 (0.72 ± 0.05), 0.72–1.00 (0.85 ± 0.10), 0.55–1.00 (0.73 ± 0.16) and 0.50–1.00 (0.76 ± 0.12) for Dice, overlap fraction, X and Y, respectively.

**Conclusion:** In our study, the overlaps between the initial high-uptake sub-volume and the recurrent metabolic volume showed moderate to good concordance. These results now need to be confirmed in a larger cohort using a more standardized patient repositioning procedure for sequential PET/CT imaging, as there is potential for RT dose escalation exploiting the pre-treatment PET high-uptake sub-volume.

## Introduction

Cervical cancer (CC) is the third most frequent cancer in the world and is responsible of more than 270,000 deaths annually (1). Diagnosis is made at a locally advanced stage in 70–80% of patients (2), for whom the standard of care consists of pelvic external beam radiotherapy (EBRT) combined with cisplatin-based chemotherapy, and followed by brachytherapy (BT) (3). However, despite recent advances in cervical cancer management especially in image-guided radiotherapy, ~40% of patients will present a recurrence after curative intent treatment, and eventually die of disease (4).

^18^F-fluorodeoxyglucose (18F-FDG) positron emission tomography/computed tomography (PET/CT) is recommended in the CC initial staging (5) or in case of recurrent disease and could play an interesting role in treatment response assessment as well (6, 7). The intensity of ^18^F-FDG uptake may also constitute a potential diagnostic and prognostic factor: pre-treatment maximum standardized uptake value (SUV_max_) has been shown to be significantly associated with the presence of synchronous lymph nodes metastasis, and to be independently correlated with recurrence and survival in a retrospective monocentric cohort of 149 patients (8). However, the role of PET for the radiotherapy planning and particularly for volume delineation has not been as extensively investigated in patients with CC as in other sites such as head and neck (9–13). Currently the determination of the gross tumor volume (GTV, corresponding to the primary tumor) and the clinical target volume (CTV) are based on magnetic resonance imaging (MRI) only. An individualized internal target volume of the tumor (ITV-T) is now also recommended based on target motion due to variable bladder and rectum filling as visualized on multiple pre-treatment CT imaging (14).

Pre-treatment high ^18^F-FDG uptake sub-volumes of tumors measured on PET/CT have been suggested as potential preferential sites of local relapse after chemoradiotherapy (CRT) in several tumor types including non-small cell lung cancer (15), rectal cancer (16), head and neck cancer (17, 18) and esophageal cancer (19) with variable levels of validation, but not in locally advanced CC (LACC) yet (20).

Our aim was to quantify the spatial overlap between recurrent metabolic disease with the more active baseline intratumoral regions in patients with LACC treated with CRT, since these metabolic tumor sub-volumes defined on the pre-treatment ^18^F-FDG PET images could then be relied upon for a more tailored approach including personalized dose distribution and/or escalation.

## Materials and Methods

### Patients

All patients with histologically proven locally advanced CC, staged IB1-IVA [FIGO 2009 definition (21)] and treated at our institution with definitive curative CRT and subsequent BT from September 2012 to December 2016 and who developed a local recurrence during the follow-up were included in this retrospective study. Available ^18^F-FDG PET/CT was mandatory. Patients with a history of chemotherapy or RT and/or metastatic disease were excluded. All patients underwent an initial ^18^F-FDG PET/CT before treatment as part of their initial staging (PET1), as well as at the time of local recurrence (PET2).

Collected data included age and date of diagnosis, histology, FIGO stage, presence of positive lymph nodes on ^18^F-FDG PET/CT, tumor size as evaluated on MRI, EBRT, and BT dose, date and site of recurrence, as well as date and status at last follow-up. Three types of recurrence were defined: local (vaginal and/or cervical), regional (pelvic/para-aortic), or distant (upper abdominal and/or extra-abdominal) (22).

All patients provided signed permission for the use of their clinical data for scientific studies and informed consent for the anonymous publication of data. Here, we considered patients with local recurrence included in a previous study that was approved by the local institutional review board approved this study (29BRC18.0015) (20, 23).

### Imaging

#### PET/CT Acquisition

Scans were performed on a Biograph mCT S64 (SIEMENS Healthineers Medical Solutions, Knoxville, TN, United States) for all patients. Standard preparation included at least 4 h of fasting and a serum blood glucose level <7 mmol/L before tracer administration. PET acquisitions were carried out ~60 min after injection of ~3 MBq (0.8 Ci)/kg of ^18^F-FDG (23).

The Biograph scanner is a 64-slice multidetector-row spiral CT with a transverse field of view of 70 cm. Standard CT parameters were used: collimation of 16 × 1.2 mm^2^, pitch 1, tube voltage of 120 kV, and effective tube current of 80 mAs. 3D PET data were reconstructed using an ordered subsets expectation-maximization (OSEM) algorithm (2 iterations, 8 subsets, TrueX 5 point spread function + time of flight) (23).

### Treatment

International guidelines were used to delineate the CTV, the planning target volume (PTV), and organs-at-risk (24). Treatment consisted of three-dimensional conformal radiotherapy (3DRT) (*n* = 15) or intensity-modulated radiotherapy (IMRT) (*n* = 6) delivered using a linear accelerator (ONCOR™ Digital Medical Linear Accelerator from Siemens® Medical Solutions, Inc or a TrueBeam STx Novalis Linear Accelerator) (23).

A pelvic EBRT or extended-field RT to the para-aortic zone using high-energy photons (18 MV), depending on the staging, at a dose of 45–50.4 Gy using standard fractionation was delivered to all patients. In patients with pelvic or para-aortic lymph node involvement, a targeted image-guided boost was delivered at a dose of 50.4–54 Gy to the affected lymph nodes (*n* = 4). Patients received 3–4 fractions of MRI-guided high dose rate (HDR) intracavitary BT every 4 days, started 1 week after the end of EBRT. The prescribed dose was 6–7 Gy for high-risk BT. The dose constraints applied were the following: CTV-HR D90 (EQD2_10_) ≥ 85 Gy, CTV-IR D90 (EQD2_10_) ≥ 65 Gy, GTV D98 (EQD2_10_) ≥ 90 Gy, D2 cm3 of bladder <90 Gy, D2 cm3 of rectum <75 Gy, and D2 cm3 of sigmoid/bowel <75 Gy (14). The ICRU (International Commission of Radiation Units) reference dose at the recto-vaginal site has to be <75 Gy. No delay or interruption of EBRT was observed due to low acute toxicity (median duration of EBRT, 49 days; range, 47–51 days). Concomitant chemotherapy consisted of 4–6 cycles of cisplatin (40 mg/m2) weekly or carboplatin (AUC 2) for patients with renal failure (23).

### Follow-up

Follow-up was performed alternatively by the radiation oncologist and the gynecologist and consisted of a physical examination every 3 months for 2 years, then twice a year until 5 years after treatment, then annually. The initial assessment 3 months after treatment was done with MRI and PET/CT, then a CT scan every 6 months until 2 years after the end of treatment was performed. A new imaging check-up was performed only if clinically indicated thereafter (23).

### Overlap

#### PET/CT Registrations

**Figure 1:**
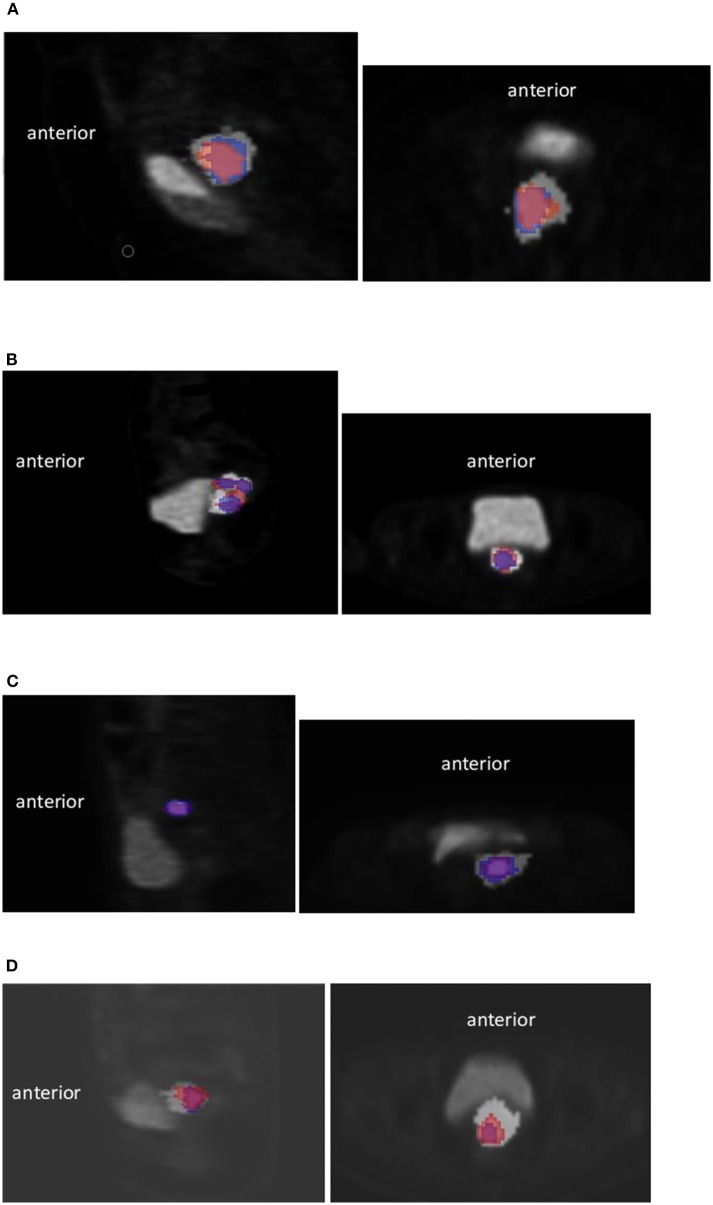
Examples of registration using the 3D Slicer TM Expert Automated Registration module in 4 different patients. Initial high 18F-FDG uptake sub-volume (V1) in blue and relapse V2 in red. Sagittal view (left) and axial (right) **(A)** for this particular case, a manual correction of the registration due to a different bladder filling was necessary (Dice = 0.73, OF = 0.77, X = 0.70, Y = 0.77), **(B)** Case with V2 larger than V1 (Dice = 0.62, OF = 0.80, X = 0.80, Y = 0.50), **(C)** Case with small V1 and V2 (Dice = 0.72, OF = 0.82, X = 0.64, Y = 0.82), **(D)** Case with V2 larger than V1 (Dice = 0.76, OF = 0.90, X = 0.90, Y = 0.65).

For each patient, a rigid registration of the pre- and recurrence-CT data sets was performed using the 3D Slicer TM Expert Automated Registration module (25) optimized with the Mattes mutual information metric (26). The transform was initialized with a registration of the two centers of mass of the images. The obtained transform was then applied to the corresponding PET. In case of obvious misalignments, manual adjustments were allowed (Figure 1 and Supplementary Material).

#### V1 and V2 Determination

Determination of volumes was carried out on PET images only. Functional uptakes derived from pre- and post-treatment (at the time of recurrence) PET (denoted as PET1 and PET2) hereinafter referred to V1 and V2 were obtained, respectively. In most previous studies (15–17, 19), only combinations of arbitrary thresholds of %SUV_max_ were used to quantify the overlaps between V1 and V2. This approach was recently highlighted as potentially leading to biased overestimated overlaps, especially when the true overlap is small or non-existent (27). It is thus advocated to rely rather on a more robust and accurate approach for the delineation of V1 and V2. We chose the fuzzy locally adaptive Bayesian (FLAB) algorithm previously validated for automatic delineation of both whole tumor and high uptake sub-volume (28, 29). Indeed, in the absence of ground-truth and based on previous results, FLAB was assumed to provide more accurate and robust volumes compared to fixed thresholds (29, 30). As in the previous study (27), FLAB was applied using 3 classes (one for the background and the other two for tumor) in PET1 to simultaneously define an overall tumor volume and V1 (the high-uptake sub-volume) (29). In PET2, FLAB was applied using 2 classes (one for background, one for tumor) in order to define V2 (31). SUV_max_, SUV_mean_ and total lesion glycolysis (TLG) values were recorded on both PET1 and PET2.

#### Overlap Analysis

The indices used to quantify the overlap between V1 and V2 were the Dice coefficient, the overlap fraction (OF) and the intersection of volumes V1 and V2 divided by V1 (X) or V2 (Y). Although they were used in most previous studies (15–17, 19), Jaccard coefficients were not included in the present work, as they provide redundant ranking with Dice.

$$\begin{array}{l} Dice=2\times \frac{V_{1}\cap V_{2}}{V_{1}+V_{2}},OF=\frac{V_{1}\cap V_{2}}{min(V_{1},V_{2)}},\\ X=\frac{V_{1}\cap V_{2}}{V_{1}},andY=\frac{V_{1}\cap V_{2}}{V_{2}}. \end{array}$$

These four metrics range between 0 and 1: very low (0–0.2); low (0.21–0.4); moderate (0.41–0.60; good (0.61–0.80), and very good concordance (0.81–1.0) (32).

## Results

### Patient and Tumor Characteristics

Twenty-one patients were included. Patients' characteristics are shown in Table 1. The mean ± SD follow-up was 26 ± 11 months. At the time of analysis, 11 patients were still alive and 10 had died from the disease. Among the 21 patients, 8 had an isolated local recurrence, 8 had local and nodal recurrences, and 5 had local and distant recurrences. As new biopsies were not performed in the 5 patients having distant metastases, pathological confirmation of the local recurrence was available in 16 patients (76%) only. All patients had a MRI and a PET (PET2) at the time of relapse. PET2 was performed 6 ± 4 months after the end of treatment.

**Table 1:** Patients' characteristics.

	***N* = 21**	**%**
Age median (range)	54 (32–79)	
FIGO stage		
IB1	1	5
IB2	1	5
IIA	1	5
IIB	12	56
IIIA	1	5
IIIB	3	14
IVA	2	10
Histology
Squamous carcinoma	17	81
Adenocarcinoma	3	14
Adenosquamous carcinoma	0	0
Clear cell carcinoma	1	5
Lymph node involvement
Uninvoled	9	43
Involved	12	57
Pelvic	8	67
pelvic and para-aortic	4	33
Treatment
3D-CRT	15	71
IMRT	6	29
EBRT dose median (range)	45 (45–54)	
BT dose median (range)	24 (21–26)	
PET1 (mean)
SUV_max_	19.5 ± 7.4	
SUV_mean_	6.6 ± 2.9	
TLG	286.4 ± 281.5g	
PET2 (mean)
SUV_max_	21.3 ± 9.9	
SUV_mean_	8.9 ± 4.1	
TLG	185.7 ± 278.4g	

*FIGO, international federation of gynecology and obstetrics; 3D-RT, three-dimensional conformal radiotherapy; IMRT, intensity-modulated photon radiotherapy; EBRT, external beam radiotherapy; BT, brachytherapy; SUV_max_, maximum standardized uptake value; SUV_mean_, mean standardized uptake value; TLG, total lesion glycolysis*.

### Registration Procedure and Tumor Volumes

A manual correction was required in 10 patients due to significant anatomical variations due to variations in bladder and/or rectum filling.

**Figure 2:**
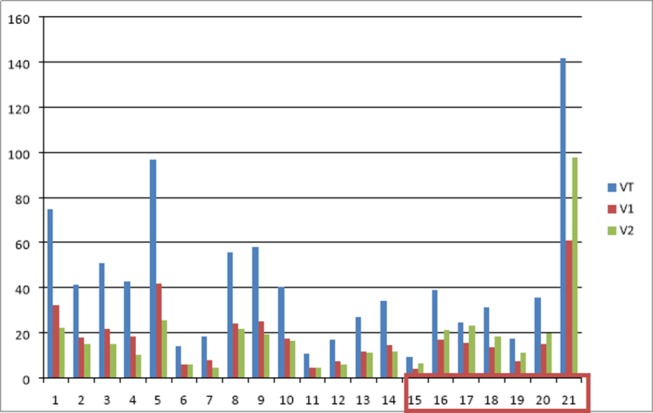
Histogram of volume values (in cc) delineated on initial and recurrence PET scans. VT is the initial tumor volume, V1 is the initial high-uptake sub-volume, and V2 is the recurrence volume (patients with V2>V1 are in the red box).

According to FLAB, the mean entire tumor volume on PET1 was 41.9 ± 31.6 cm^3^ and was significantly larger than the mean high-uptake sub-volumes (V1) of 18.2 ± 13.5 cm^3^ (*p* < 0.01). V2 volumes on PET2 were 15.1 ± 13.5 cm^3^ (Figure 2). Seven patients (33%) had a V2 larger than V1. As noted earlier, in cases where the volume of recurrence is measured as greater than the high uptake pre-treatment subvolume, the overlap analysis is no longer relevant (27). In these situations, V2 would likely include a large part (or even the whole) of pre treatment volume and most likely all of the highly absorbent sub-volume, resulting in biased overlap measures. It would also be unnecessary to use this candidate PET sub-volume for dose escalation, as this would either mean increasing the total pre-treatment volume or a sub-volume that would not cover the overall relapse volume (V2) (27).

Mean SUV_max_, SUV_mean_ and TLG values were 19.5 ± 7.4, 6.6 ± 2.9, and 286.4 ± 281.5 g on PET1 and 21.3 ± 9.9, 8.9 ± 4.1, and 185.7 ± 278.4 g on PET2, respectively (Table 1, Supplementary Material). There were no significant differences between values on PET1 and PET2.

### Overlaps Between the Initial High-Uptake Sub-Volume (V1) and Recurrence Volume (V2)

A good overlap was found between the initial high-uptake sub-volume (V1) and recurrence volume (V2) for the 21 patients. median values for Dice, OF, X and Y were 0.73 (range, 0.62–0.81), 0.82 (range, 0.72–1.00), 0.70 (range, 0.55–1.00), and 0.75 (range, 0.50–1.00).

**Figure 3:**
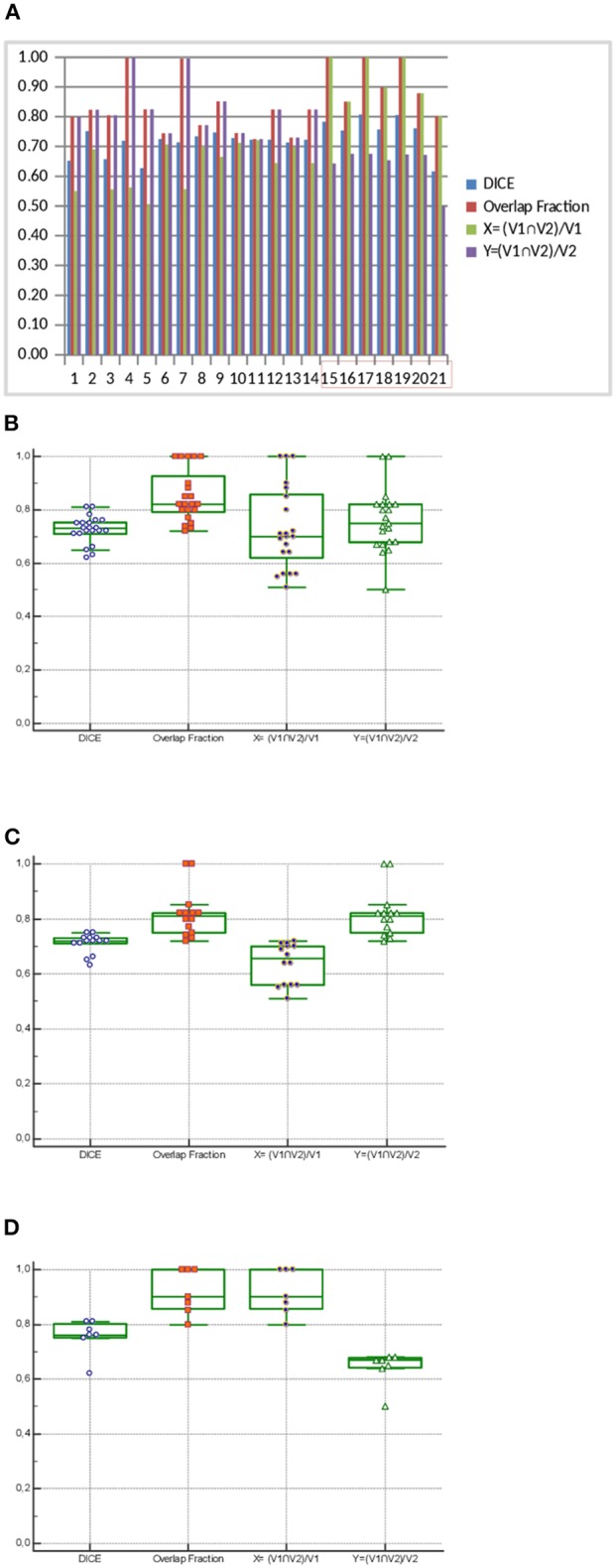
Histogram (**A**, patients with V2>V1 are in the red box) and box plot corresponding (**B**: *n* = 21 including 7 patients with V2 < V1, **C**: *n* = 14 V2 < V1, and **D**: *n* = 7 V2>V1) to the overlap analysis: Dice coefficient, Overlap fraction, and intersection of V1 and V2 volumes divided by either V1 (X = (V1nV2)/V1) or V2 (Y = (V1nV2)/V2).

As expected, the 4 metrics were slightly different when excluding the 7 patients with a higher V2 than V1, with high and consistent values for all 4 metrics. The median values for Dice, OF, X, and Y were 0.72 (range, 0.63–0.75) (Figure 3), 0.81 (range, 0.73–1.00), 0.65 (range, 0.51–0.71), and 0.81 (range, 0.72–1.00), respectively.

## Discussion

Our study suggested that the initial high-uptake sub-volume on baseline PET/CT images is at higher recurrence risk. These areas should be included in the high risk CTV during BT and their identification could also allow for an additional PET-guided boost during EBRT.

To our knowledge, no other studies have shown that FDG hotspots on pretreatment FDG PET/CT can detect tumor areas at high risk of recurrence in locally advanced cervical cancers. Our results concur with previous studies in others cancers sites (15–17, 19). Indeed, two recents studies conducted by Calais et al. (19, 33) tested several combinations of threshold values and showed that the overlaps were estimated to be sufficiently high to justify radiotherapy planning optimization in patient with NSLSC and esophageal cancer. In 17 patients with a local relapse of lung cancer, the subvolumes defined on pretreatment PET/CT scans with 70–90% SUV_max_ thresholds were in good overlap with the recurrent volume at a 40% SUV_max_ threshold (OF indices 0.60–0.80) (33).

In 35 patients with a local relapse of esophageal cancer, the subvolumes delineated on initial PET/CT with a 30–60% SUV_max_ threshold were in good to excellent agreement with the relapse volume using a threshold of 90% SUV_max_ (OF indices 0.61–0.89) (19). Authors therefore suggested to use a 70% (for lung tumor) and 60% (for esophageal tumor) SUV_max_ threshold to identify subvolume of high ^18^F-FDG uptake on pretreatment PET/CT scans as the target areas for potential radiotherapy dose boosting. With this hypothesis, Thureau et al. (34) has recently assessed the feasibility of a FDG PET-guided dose escalation with IMRT in 21 non squamous cell lung carcinomas (RTEP5 trial, NCT01576796). In using a boost to FDG hotspot delineated with 70% SUV_max_ threshold on primary tumor, the mean dose to planning target volume was 72.5 ± 0.25 Gy and the dose/volume (D/V) constraints to organ at risk (OAR) were respected.

Similar findings using the same approach were also obtained in 24 patients with rectal cancer, although elastic transform was used to register PET/CT images, which is likely to have biased the overlap analysis by deforming tumor volumes (16).

In two other studies focusing on head and neck cancer, the overlaps were lower and showed only a moderate agreement [0.52–0.43 in 19 patients (17) and 0.52–0.39 in 38 patients (18)]. These findings were attributed to sub-optimal registration and the lack of contention device and positioning protocol to harmonize pre-treatment and post-treatment acquisitions. It is also probably explained by the upper aero-digestive tract anatomic subsite that is strongly impacted by treatment in terms of tissues distortions, making the registration process more difficult. Beaumont et al. (35) recently proposed to use voxel to voxel radiomic analysis to improve LR sites prediction. They showed that a combination of 15 textural and spatial location parameters allowed a better prediction of the GTV failure than a regional analysis, although 11 patients out of 26 were not analyzed due to lack of accurate registration and only cross-validation could be performed.

However, it should be emphasized that most of these studies used combinations of arbitrary fixed thresholds. Fixed thresholding of PET intensities has been shown to lack both accuracy and robustness (28, 30). Such an approach can lead to artificially overestimate (or underestimate) overlap, especially when it is actually small or even non-existent (27). For instance, a 70 or 90% SUV_max_ threshold is likely to generate very small volumes that usually underestimate the true size of the residual uptake, while a threshold of 40% may not be appropriate either, resulting in an overestimation of the uptake, especially for small, low-contrast uptakes (36). This issue of biased overlap estimation adds to the already important physical (PET1 and PET2 registration issues) and biological (the actual biological overlaps between high uptake pre-treatment sub-volumes and the areas of future recurrence after treatment,) uncertainties. In order to rigorously investigate this hypothesis, it is therefore crucial to rely on more robust and accurate determination of functional volumes in both pre-treatment and post-treatment PET images. To accomplish this, a number of automatic methods have been validated and all of them can provide more accurate results for V2 compared to fixed thresholds. The determination of V1 is however more complex, since the aim is to delineate a high-uptake sub-volume instead of the entire uptake. This is why we chose FLAB for the present work since it has been developed and rigorously validated specifically for the task of determining automatically and simultaneously both the entire volume and the high uptake tumor sub-volume (29). The definition of the overall residual uptake in PET2 using 2 classes also provided a more robust and accurate definition of its spatial extent, given the demonstrated robustness of FLAB for smaller and lower contrast uptakes in comparison of fixed thresholds approaches (28, 31). In this study, we used a validated automatic segmentation method to reduce manual input and increase consistency. Although FLAB is not freely available, other equally efficient PET segmentation tools are available in clinical routine, such as improved adaptive thresholding (37) or gradient-based method (36), so our results could be reproduced by others.

Our study has several limitations. It is monocentric and retrospective, with a small number of patients. However, local relapse after treatment for locally advanced CC are not common. We also included 13 patients with local recurrence along with distant or regional recurrences, and this could bias the results. Indeed, the primary tumor recurrence may have a different biological behavior when it is associated with metastases (38), and this could translate into different imaging characteristics. Additionally, only primary tumors were analyzed. Moreover, despite the use of a validated automatic registration method [the 3D Slicer TM Expert Automated Registration module (25) optimized with the Mattes mutual information metric (26)], manual correction was required in 10 patients due to significant anatomical variations related to variations in bladder and/or rectum repletion. These corrections can lead to intra- or inter-observer variabilities.

Dice coefficients are more sensitive to the differences in sizes of the two compared volumes, whereas OF leads usually to higher values due to the use of the smallest volume in the denominator. This means that “valid” values can be obtained for these indices even if the volumes being compared are not accurate and their overlap is not spatially valid (in terms of absolute volume or location/form) (27). But in our study V1 and V2 were not very different, which limits the risk of overestimation. We also included 2 other indices, X and Y, to minimize this bias and the four metrics provided a rather consistent evaluation of the overlaps.

Another important bias are the volumes: if functional uptakes derived from pre-treatment PET are larger than those derived from post-treatment PET, it may bias at least partly the reported overlaps toward artificially higher values. In our cohort, seven patients (33%) had a V2 larger than V1.These patients had the same treatment characteristics and outcome than the other 14 patients with V2 < V1. Our results still show a good overlap between V2 and V1 after excluding them.

Beyond these potential sources of bias, identifying ^18^F-FDG hotspots on initial ^18^F-FDG PET/CT is a promising approach for personalized treatment in patients undergoing CRT, but, actually, it can not be implemented in clinical practice. Indeed, limitations regarding the reproducibility and robustness of the process has to be improved.

Our previous results suggest that 2 radiomic features in ^18^F-FDG PET and in ADC maps from Diffusion-weighted MRI (DWI MRI) are highly predictive of the efficacy of CRT in the treatment of CC (23, 39). Indeed, high values of these parameters are associated with worse outcome, confirming that more heterogeneous tumors have a poor prognosis. These results can be used to personalize treatment. The present work provides additional information and BT dose escalation to pre-therapeutic identified hotpots in patients at high risk of isolated loco-regional relapse could be investigated in clinical trials.

## Conclusion

Areas with high ^18^F-FDG uptake on pretreatment PET/CT can identify tumor sub-volumes that are at high risk for recurrence in patients with locally advanced cervical cancer treated with concomitant chemoradiotherapy followed by brachytherapy. The identification of such ^18^F-FDG hotspots could justify a personalized targeted treatment in the future to decrease the risk of local recurrence. Further larger prospective studies are needed to confirm and externally validate these observations.

## Data Availability Statement

The datasets generated for this study are available on request to the corresponding author.

## Ethics Statement

The studies involving human participants were reviewed and approved by Comité éthique Centre Hospitalier Universitaire de Brest. The patients/participants provided their written informed consent to participate in this study.

## Author Contributions

FL: clinical experiment in cervical cancer and PET, study design, image analysis, statistical analysis, manuscript writing/editing. OM: clinical experiment, manuscript writing/editing. RA: experiment in PET using, manuscript writing/editing. VB, GD, and OP: clinical experiment in cervical cancer and PET, manuscript writing. MH: experiment in PET using, statistical analysis, manuscript writing/editing, image analysis. US: clinical experiment in cervical cancer and PET, manuscript writing/editing.

## Conflict of Interest

The reviewer CR declared a past co-authorship with one of the authors MH to the handling Editor. The remaining authors declare that the research was conducted in the absence of any commercial or financial relationships that could be construed as a potential conflict of interest.

## Abbreviations

BT: brachytherapy
CC: cervical cancer
CRT: chemoradiotherapy
CTV: clinical target volume
FLAB: fuzzy locally adaptive Bayesian
GTV: gross tumor volume
ITV-T: internal target volume of the tumor
IMRT: intensity-modulated radiotherapy
MRI: magnetic resonance imaging
OF: overlap fraction
OAR: organ at risk
OSEM: ordered subsets expectation-maximization
RT: radiotherapy
SUV_max_: maximum standardized uptake value.
