# Supplementary material for: Use of Baseline 18F-FDG PET/CT to Identify Initial Sub-Volumes Associated With Local Failure After Concomitant Chemoradiotherapy in Locally Advanced Cervical Cancer
Source: Front Oncol. 2020 May 7;10:678. doi: 10.3389/fonc.2020.00678 (PMC7221149; doi:10.3389/fonc.2020.00678)
Supplement: Supplementary file 1 [file Data_Sheet_1.docx]

**Figure S1:** Examples of registration using the 3D Slicer TM Expert Automated Registration module in 4 different patients. The initial tumor volume (VT) in grey,initial high 18F-FDG uptake sub-volume (V1) in blue and relapse V2 in red. Sagittal view (left) and axial (right) (A) for this particular case, a manual correction of the registration due to a different bladder filling was necessary (Dice=0.73, OF=0.77, X=0.70, Y=0.77), (B) Case with V2 larger than V1 (Dice=0.62, OF=0.80, X=0.80, Y=0.50), (C) Case with small V1 and V2 (Dice=0.72, OF=0.82, X=0.64, Y=0.82), (D) Case with V2 larger than V1 (Dice=0.76, OF=0.90, X=0.90, Y=0.65)

A.


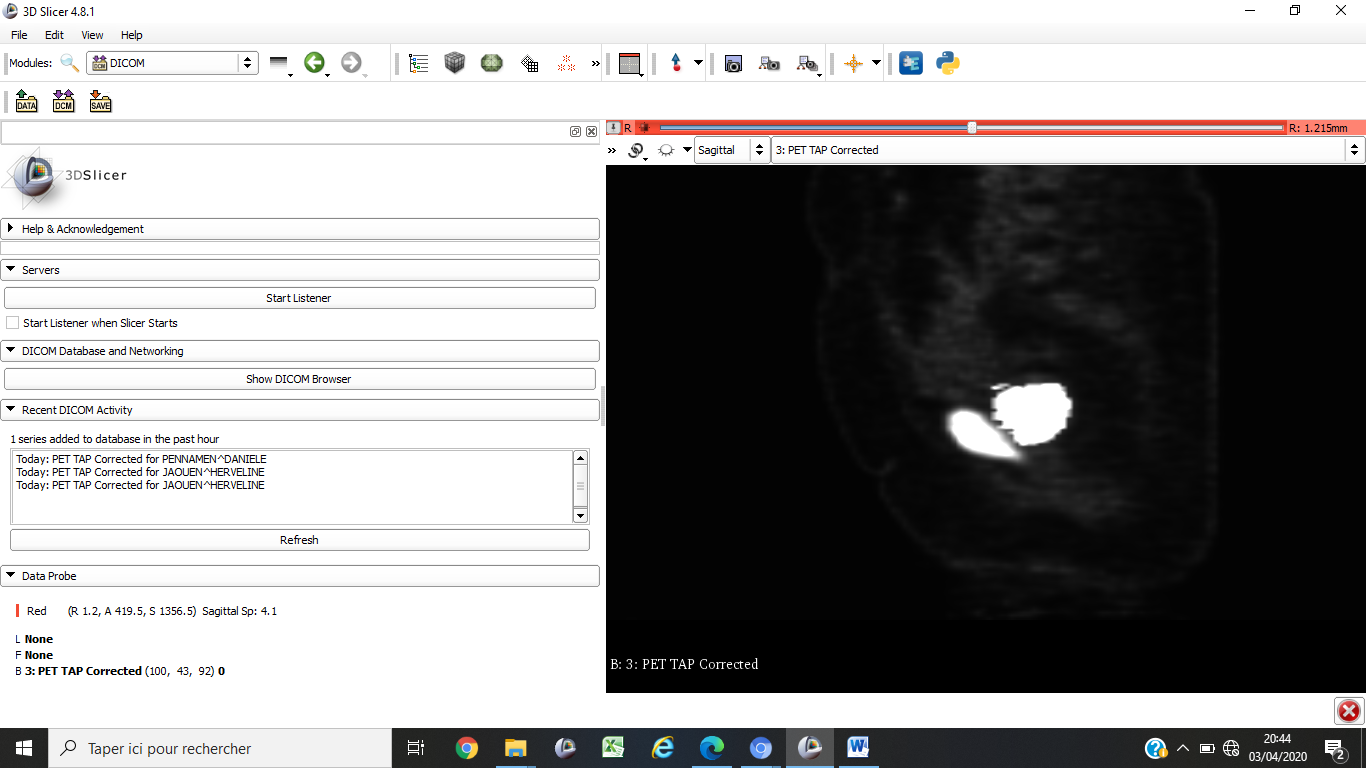

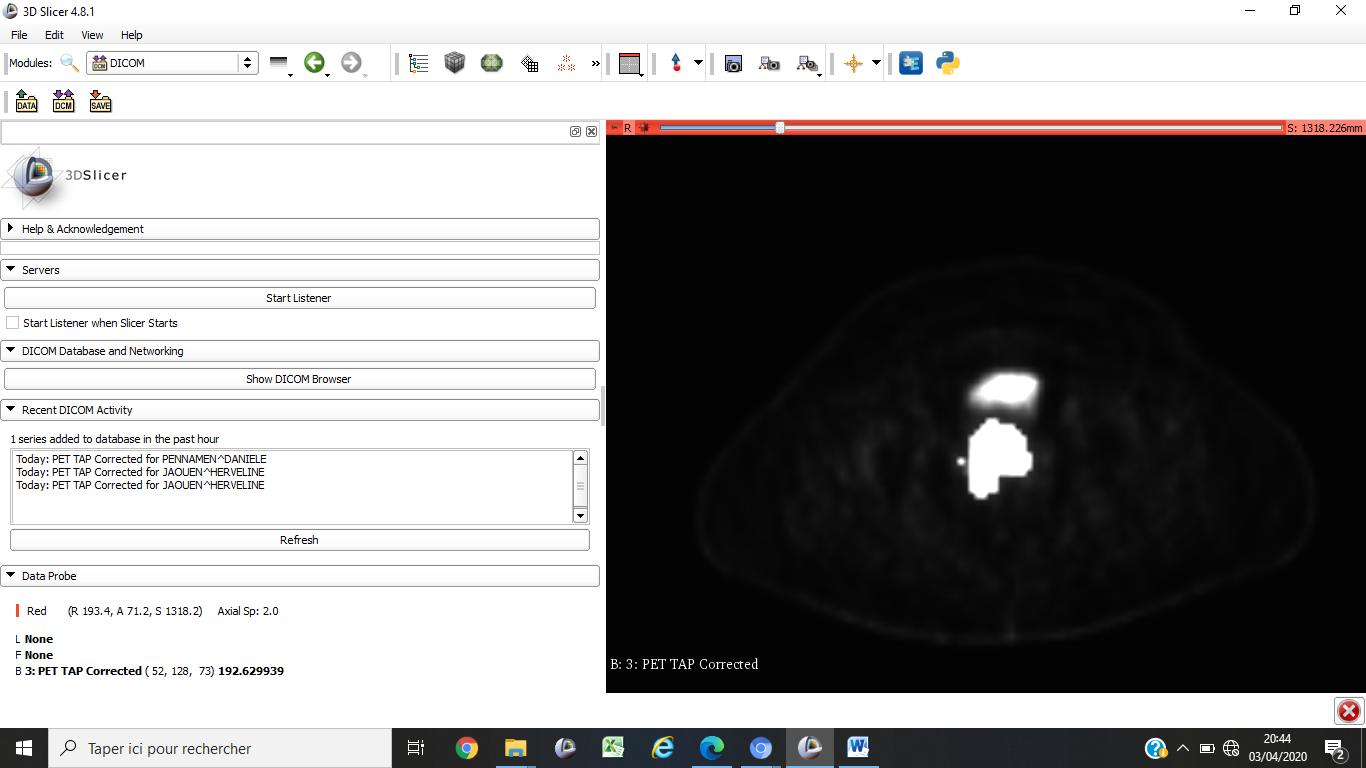


anterior

anterior


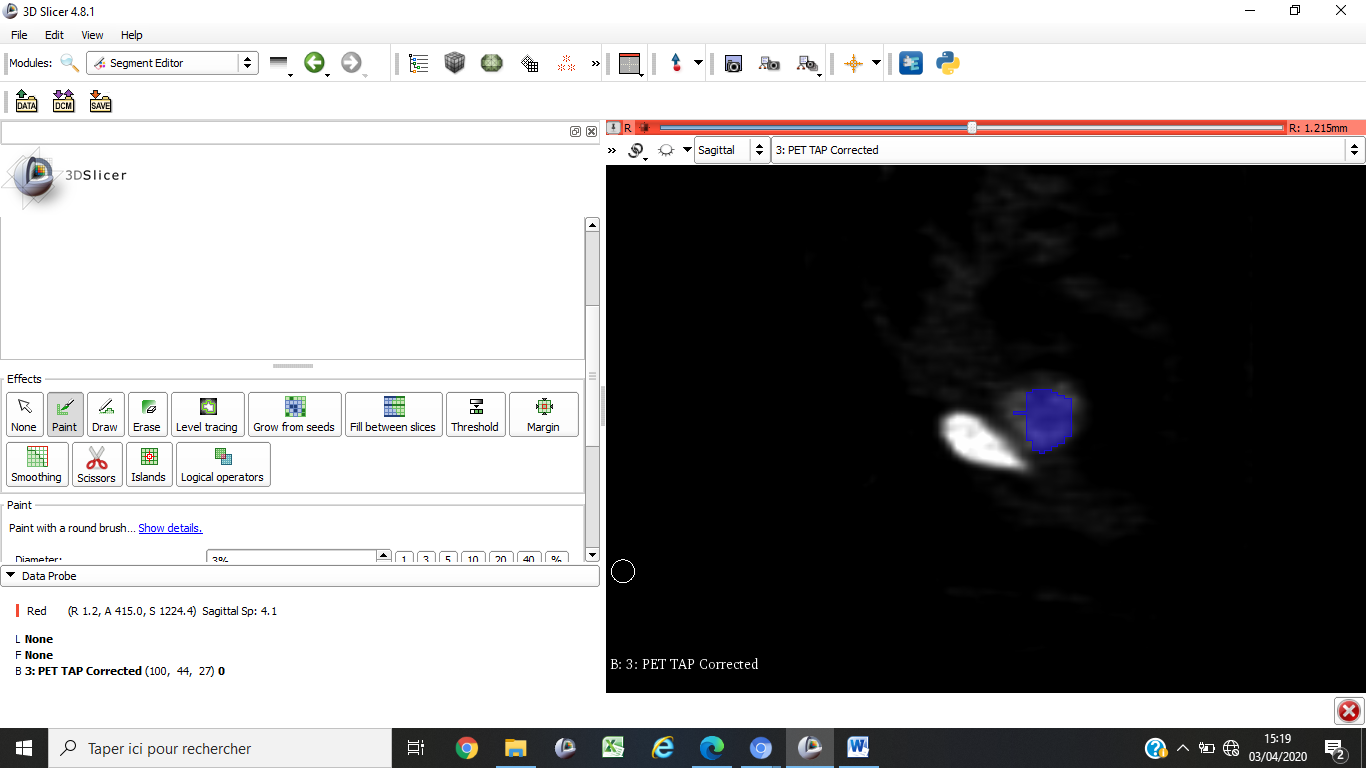

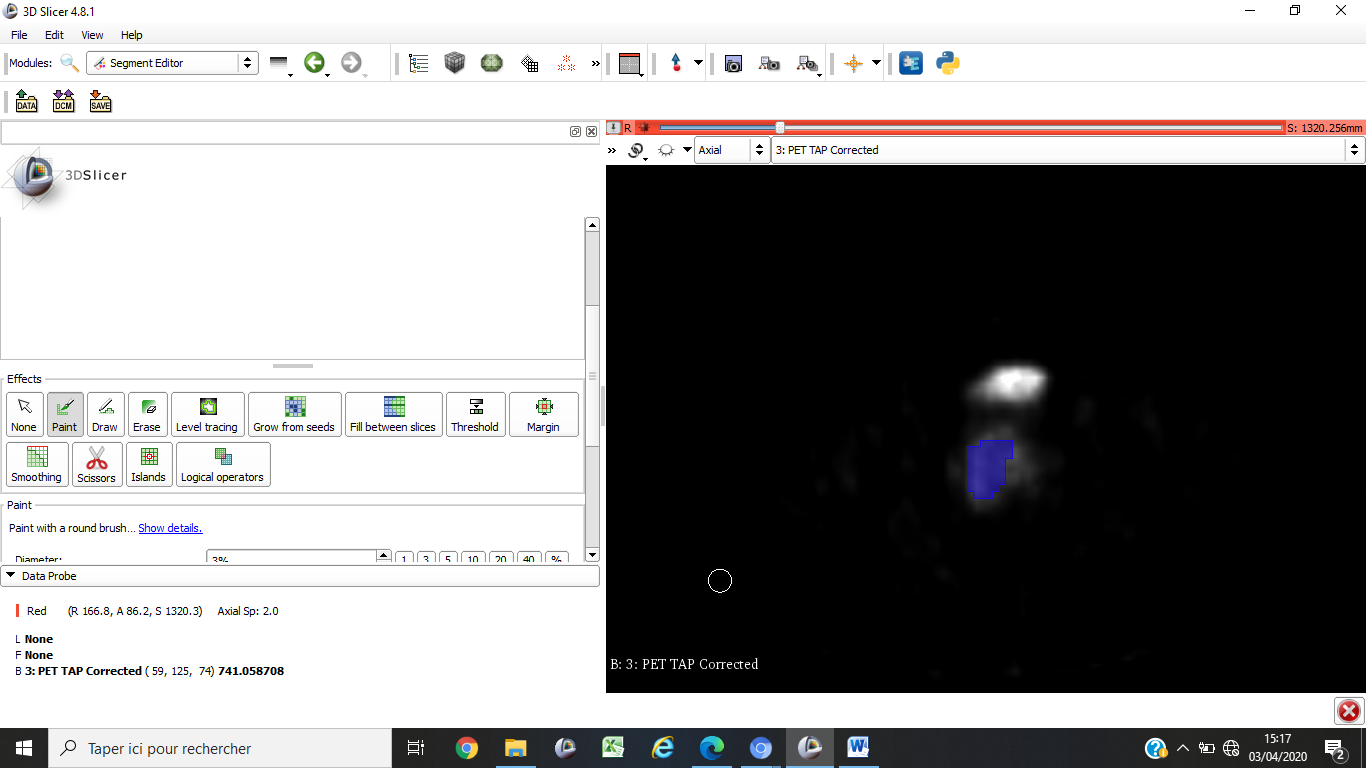


anterior

anterior


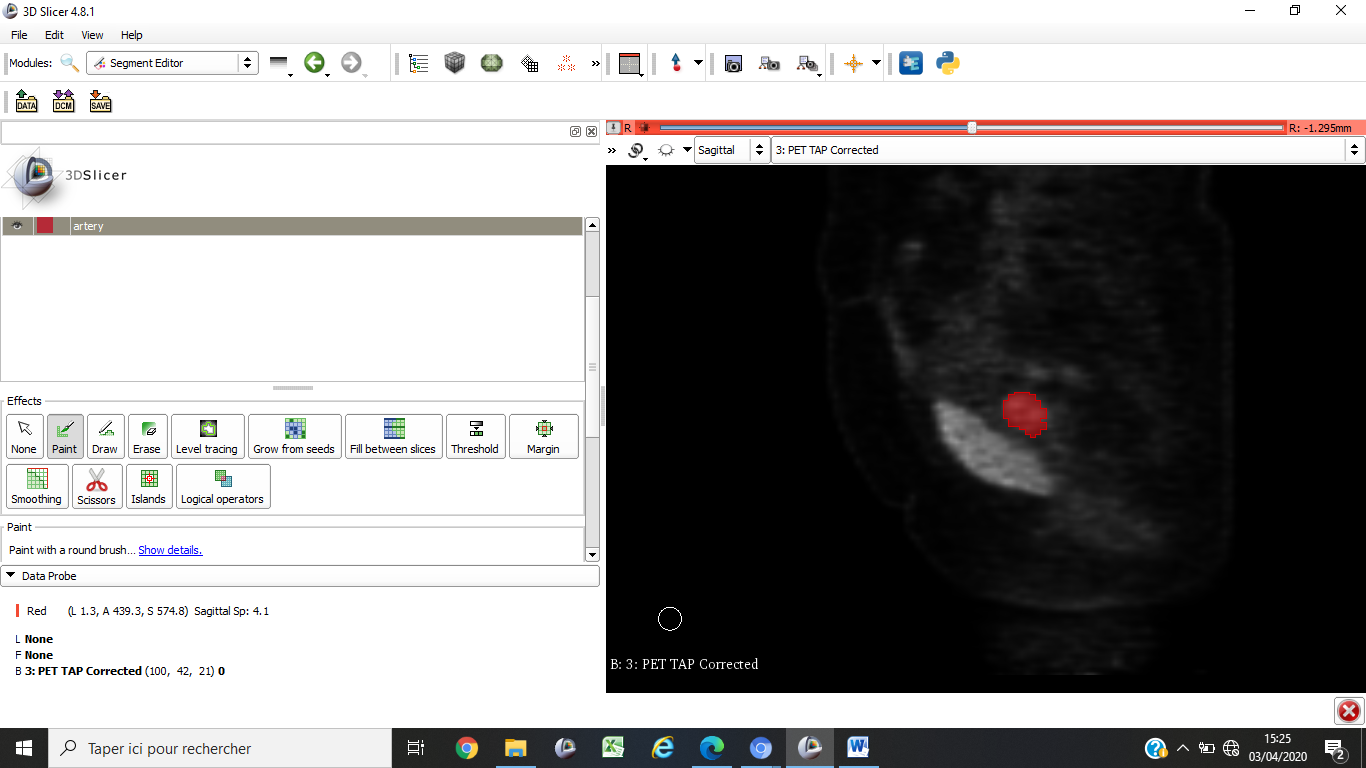

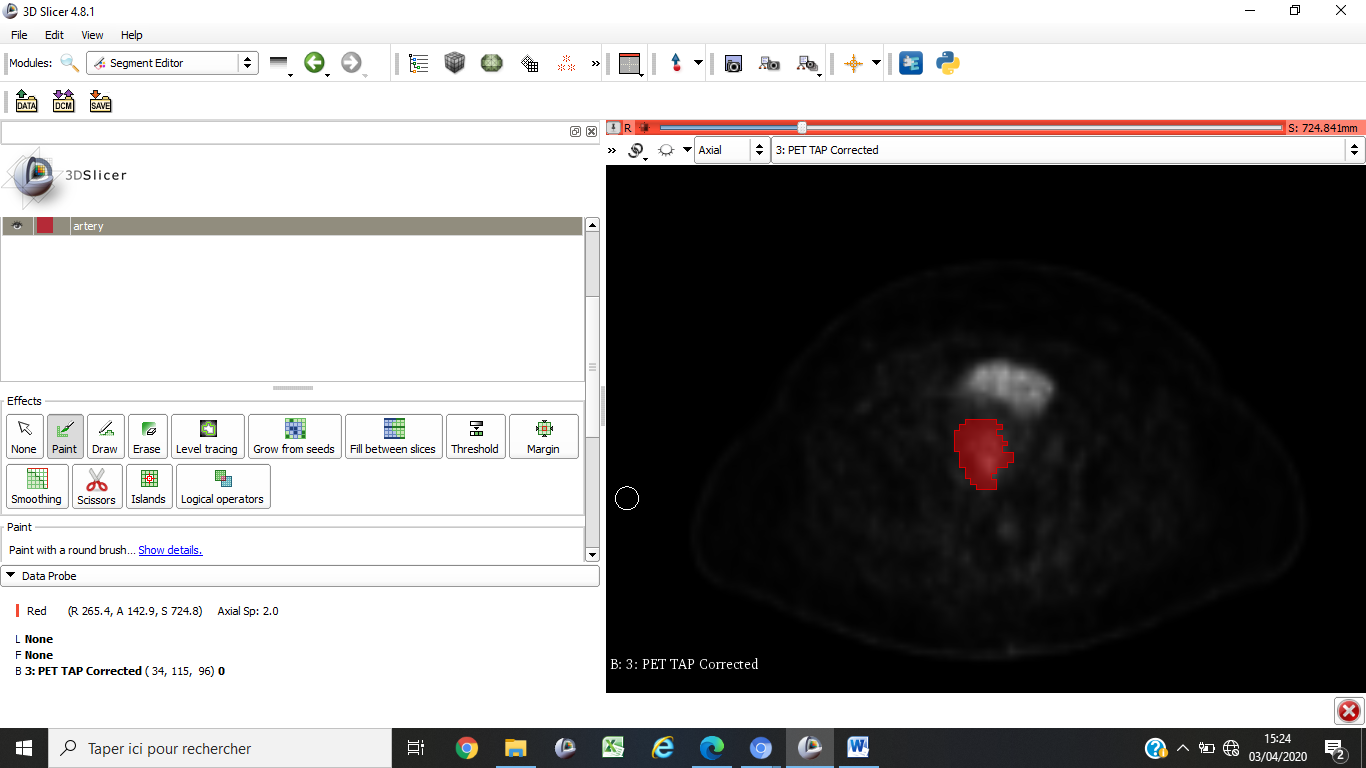


anterior

anterior


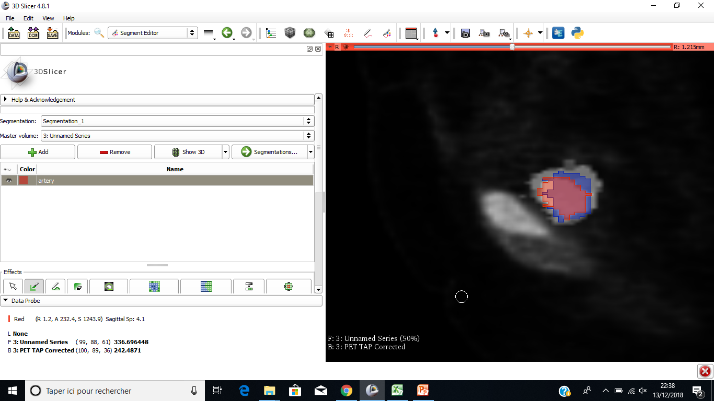

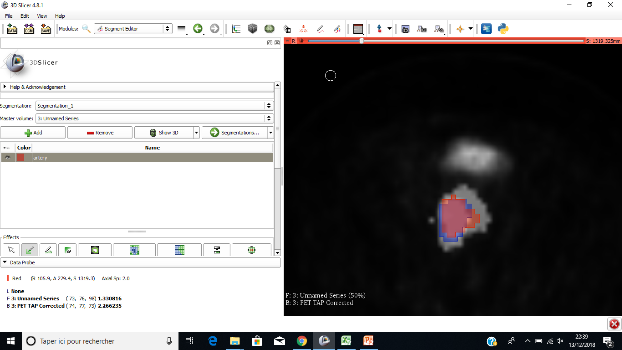


anterior

anterior

B.


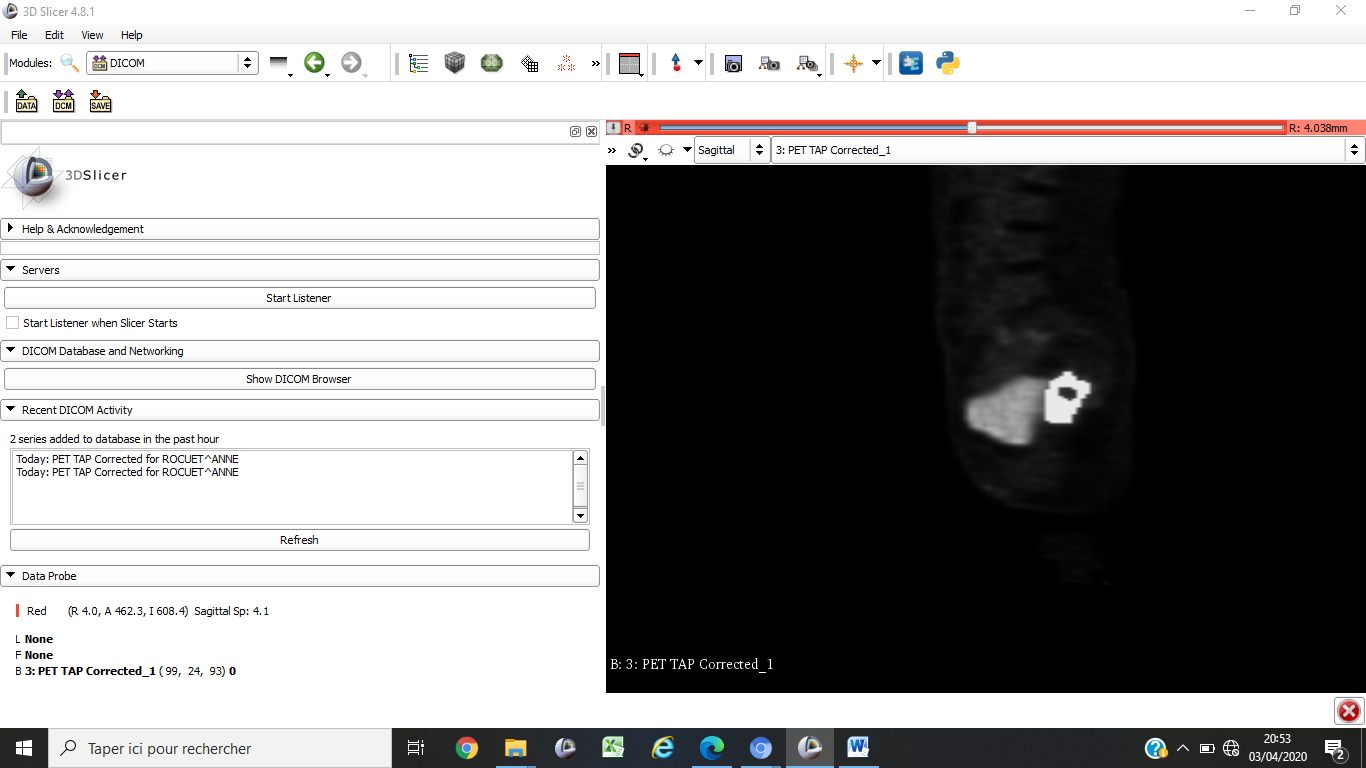

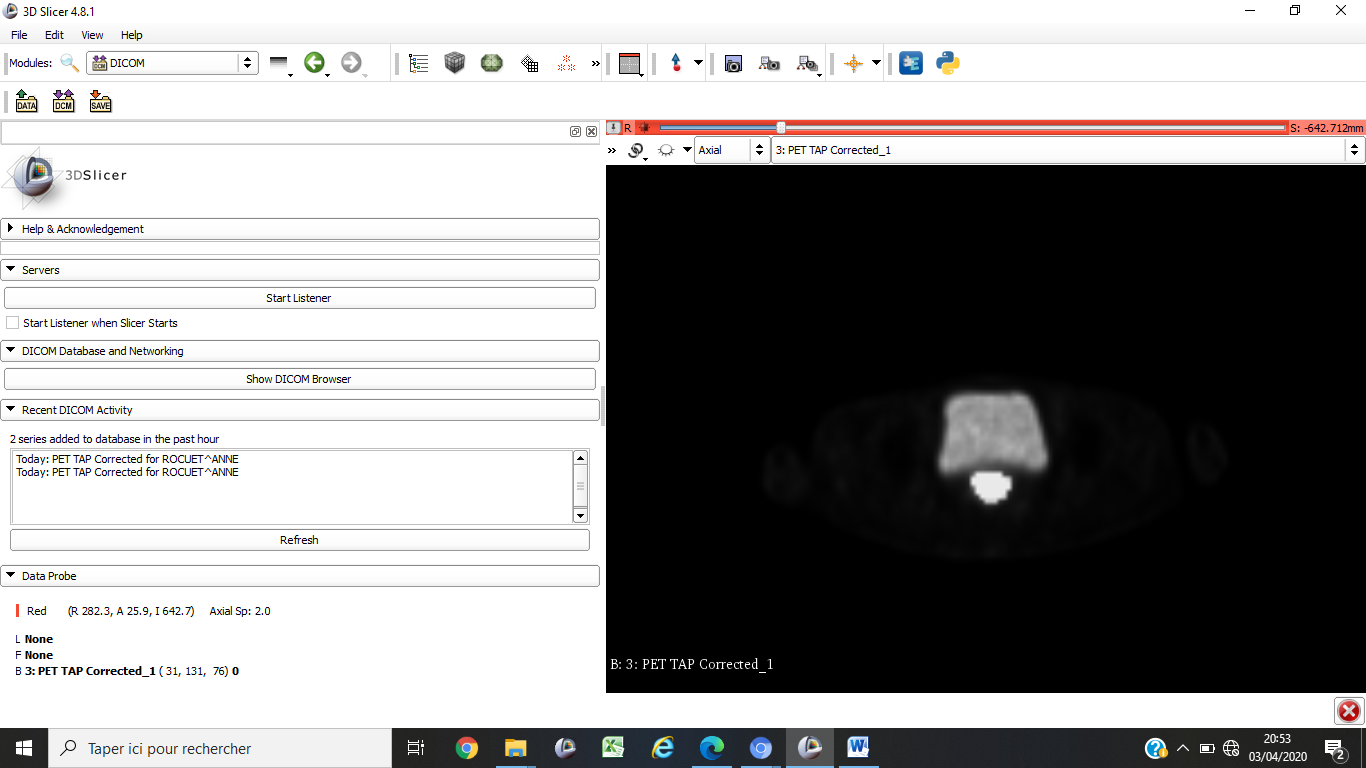


anterior

anterior


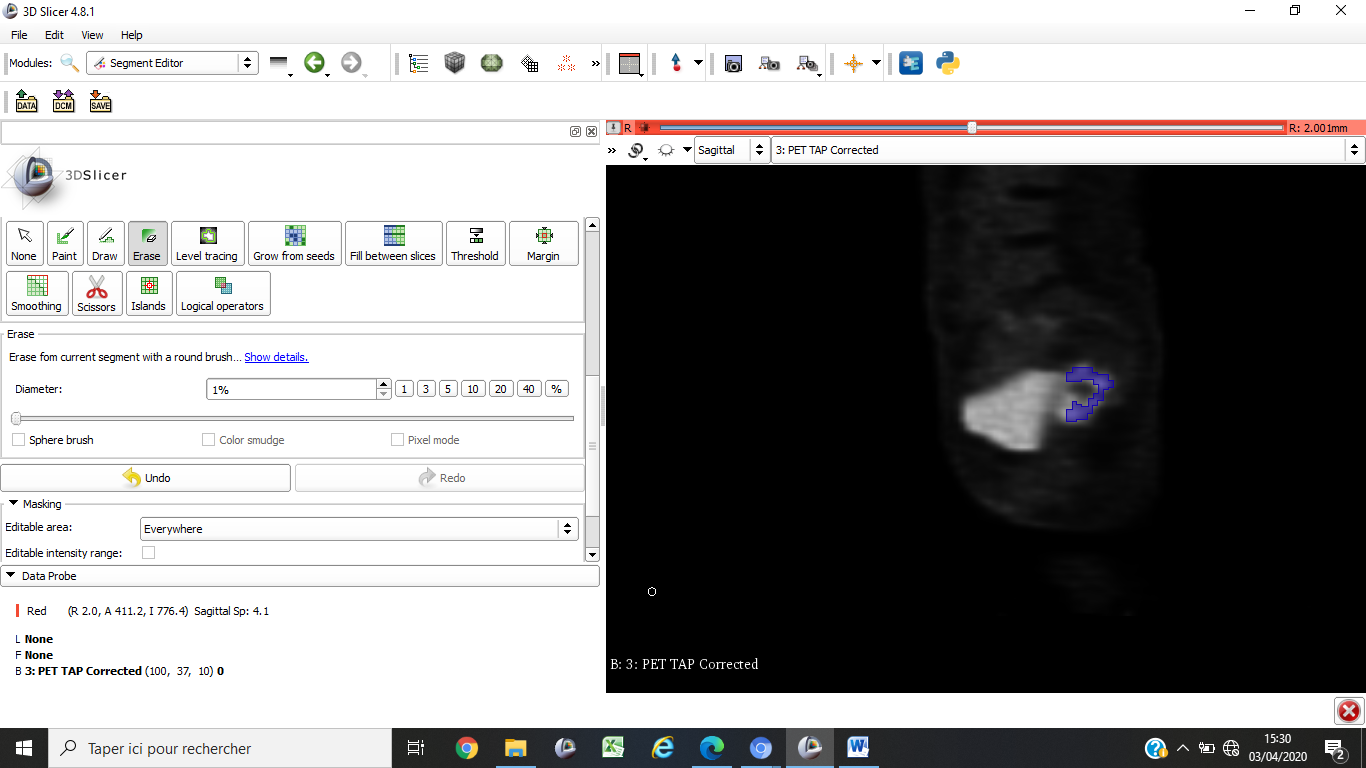

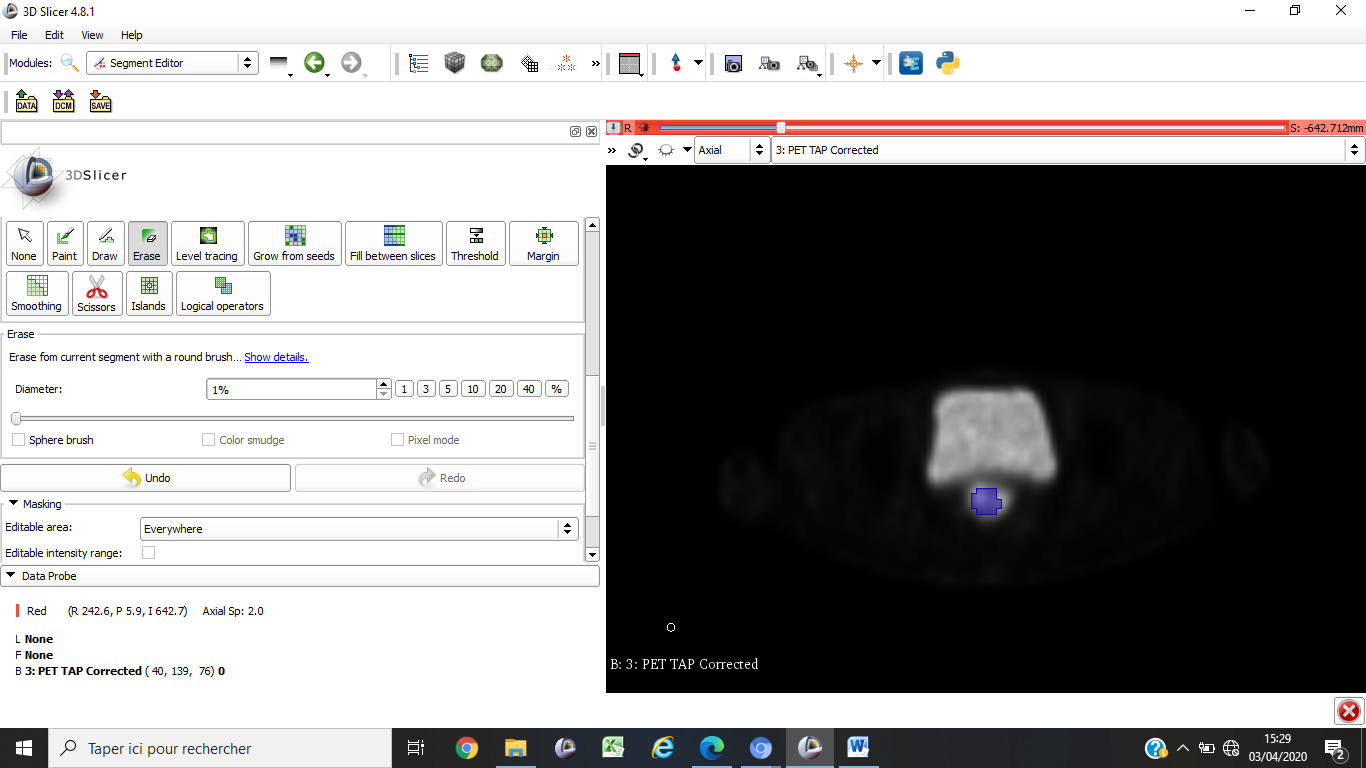


anterior

anterior


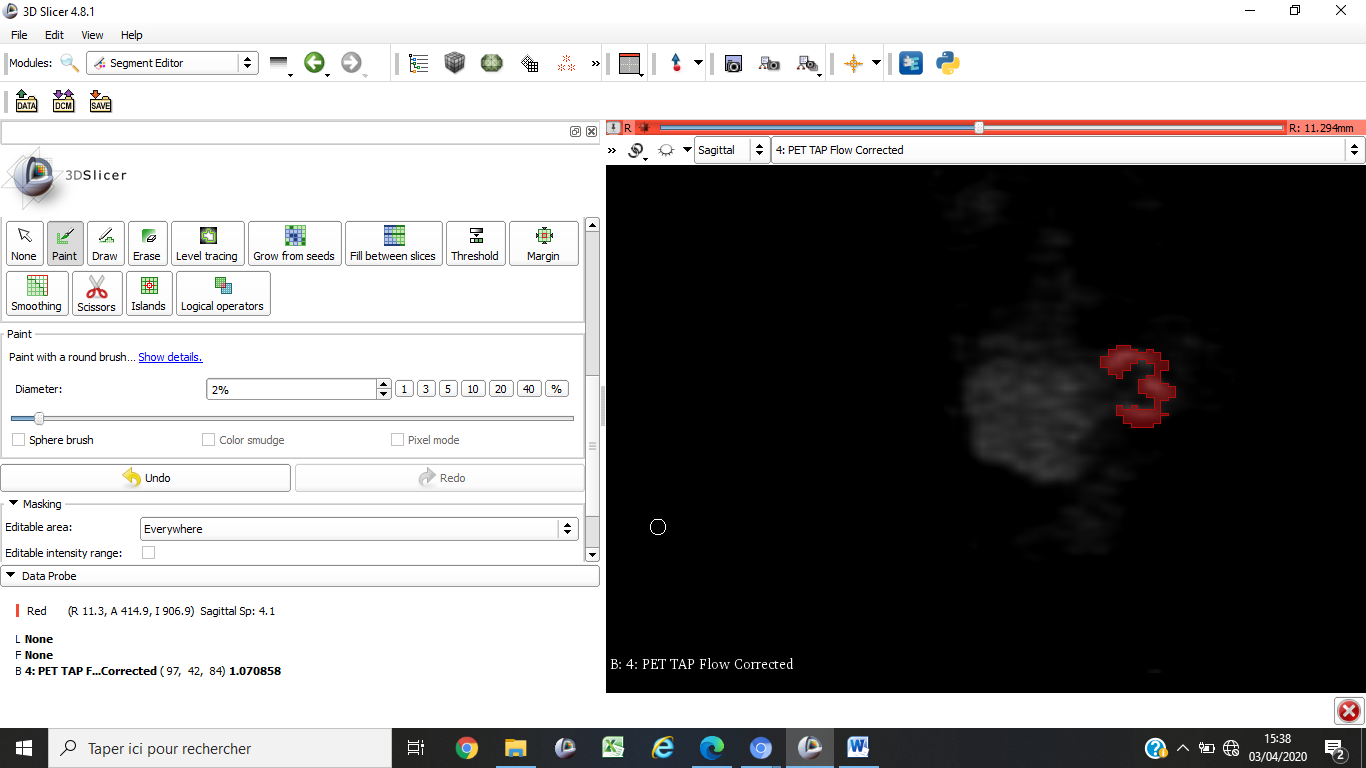

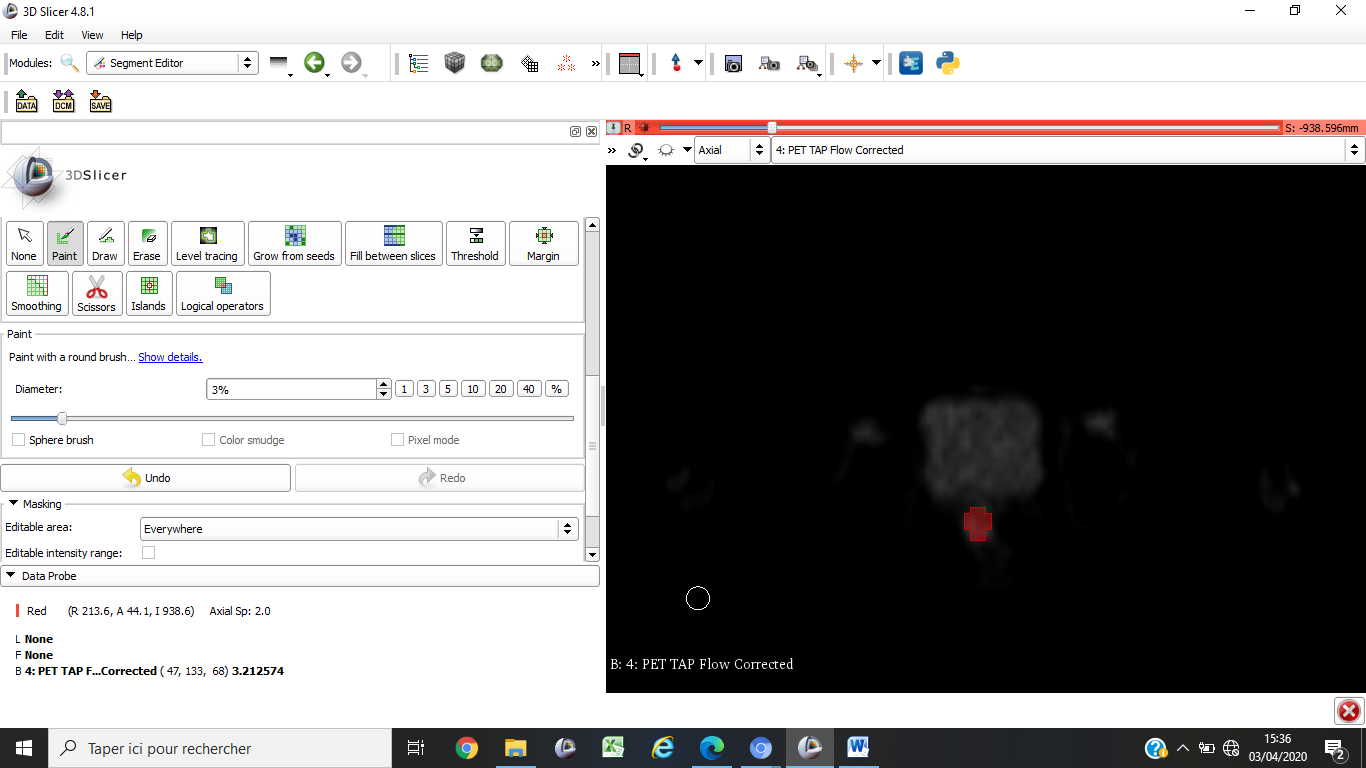


anterior

anterior


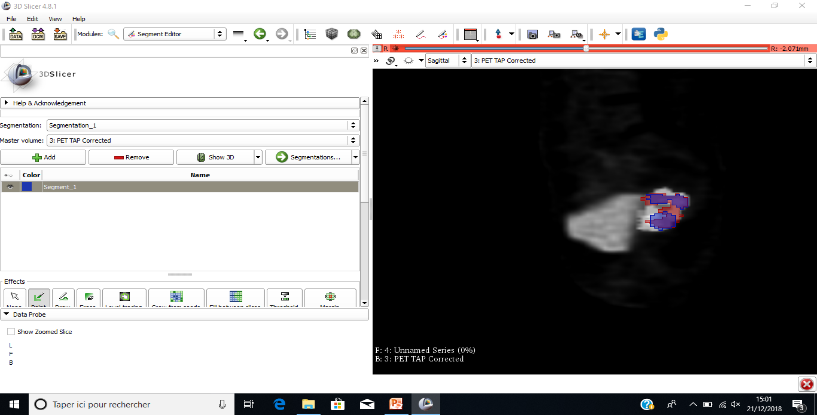

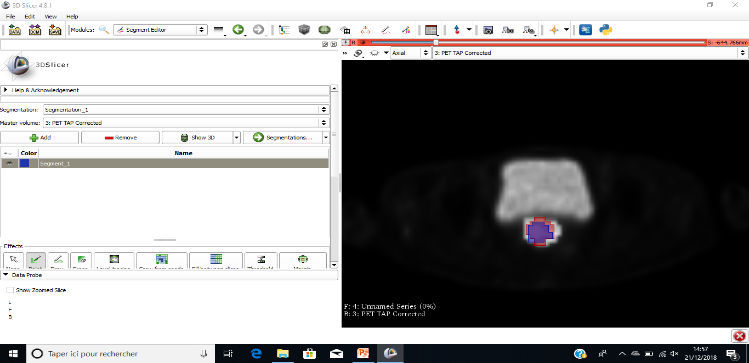


anterior

anterior

C.


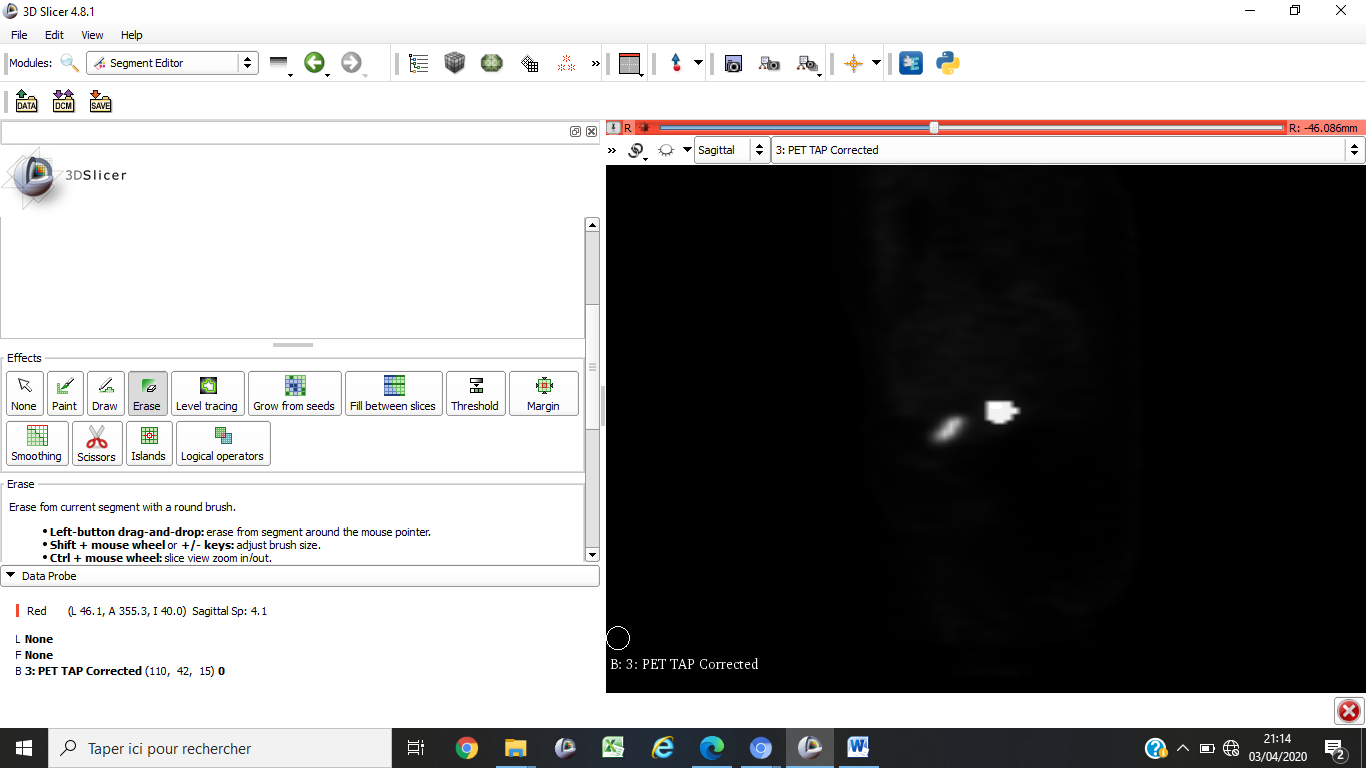

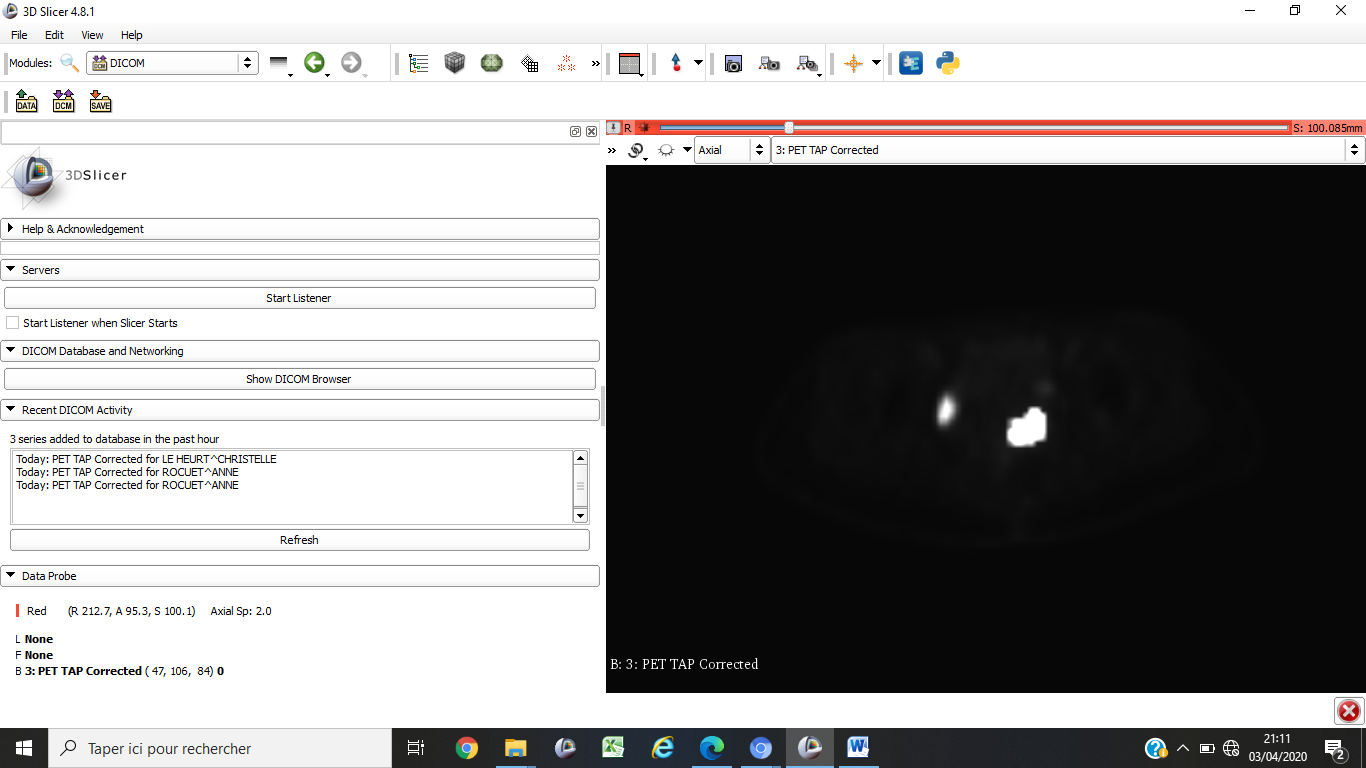


anterior

anterior


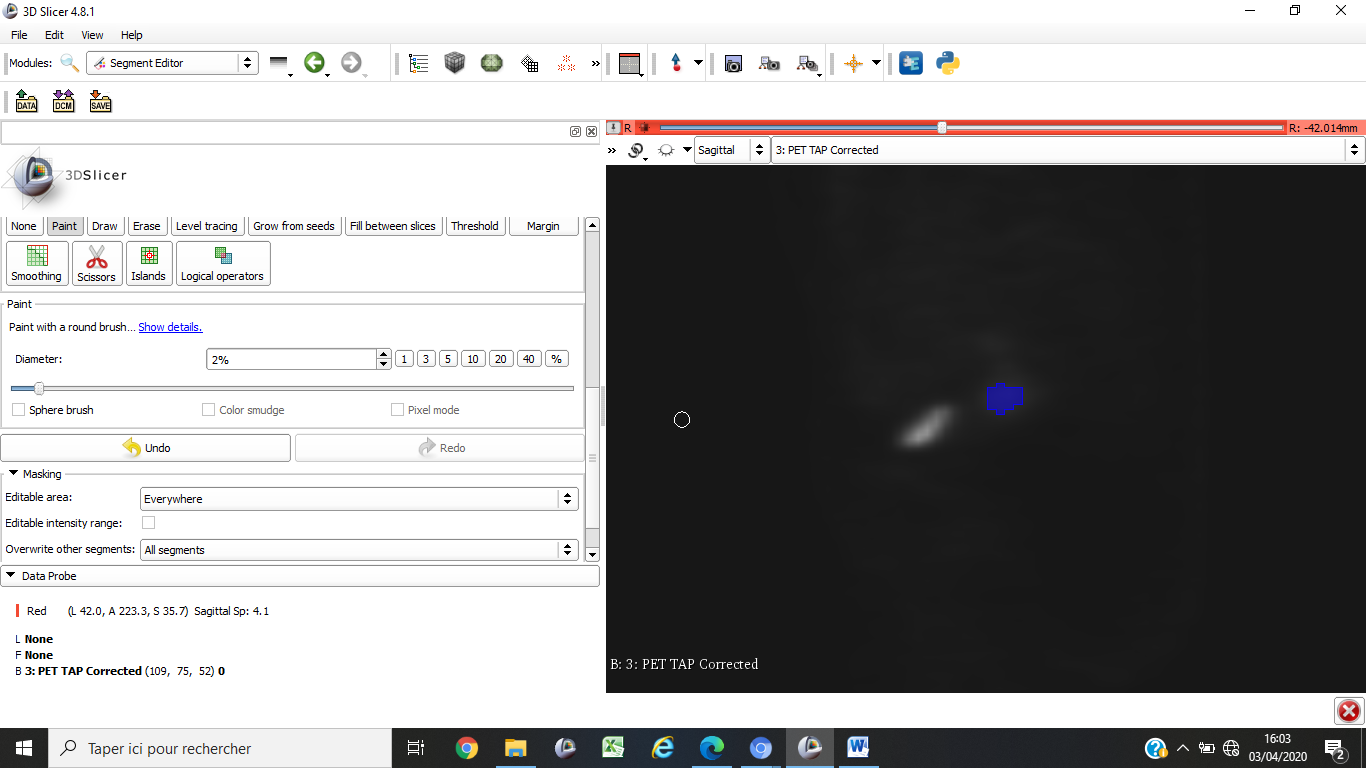

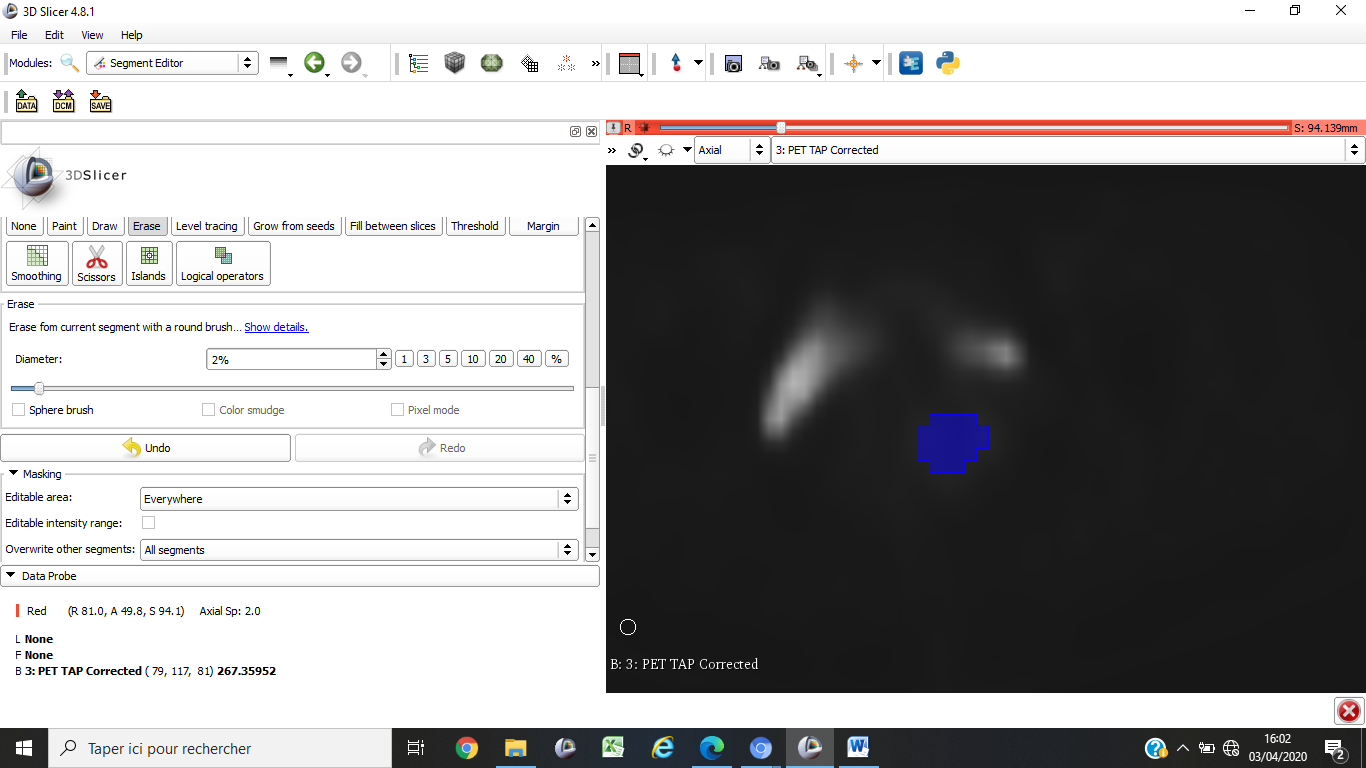


anterior

anterior


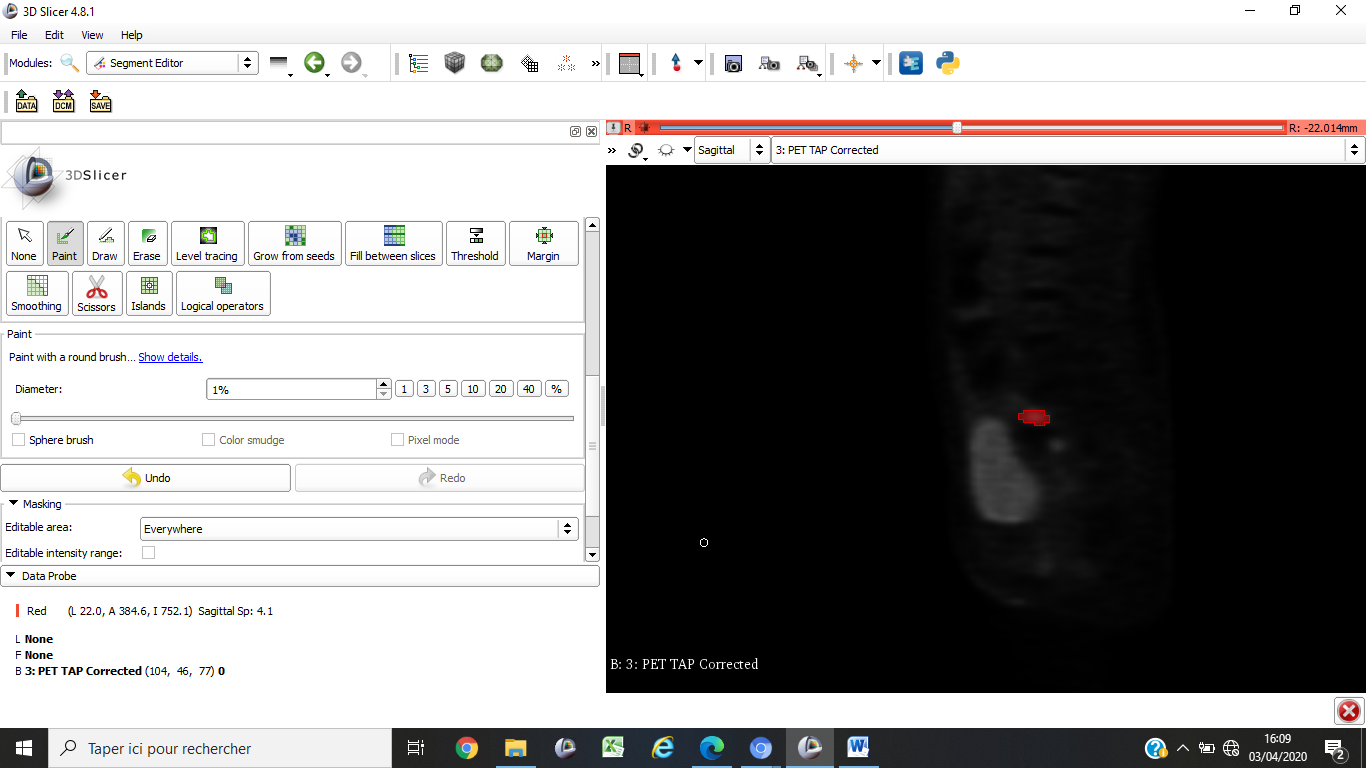

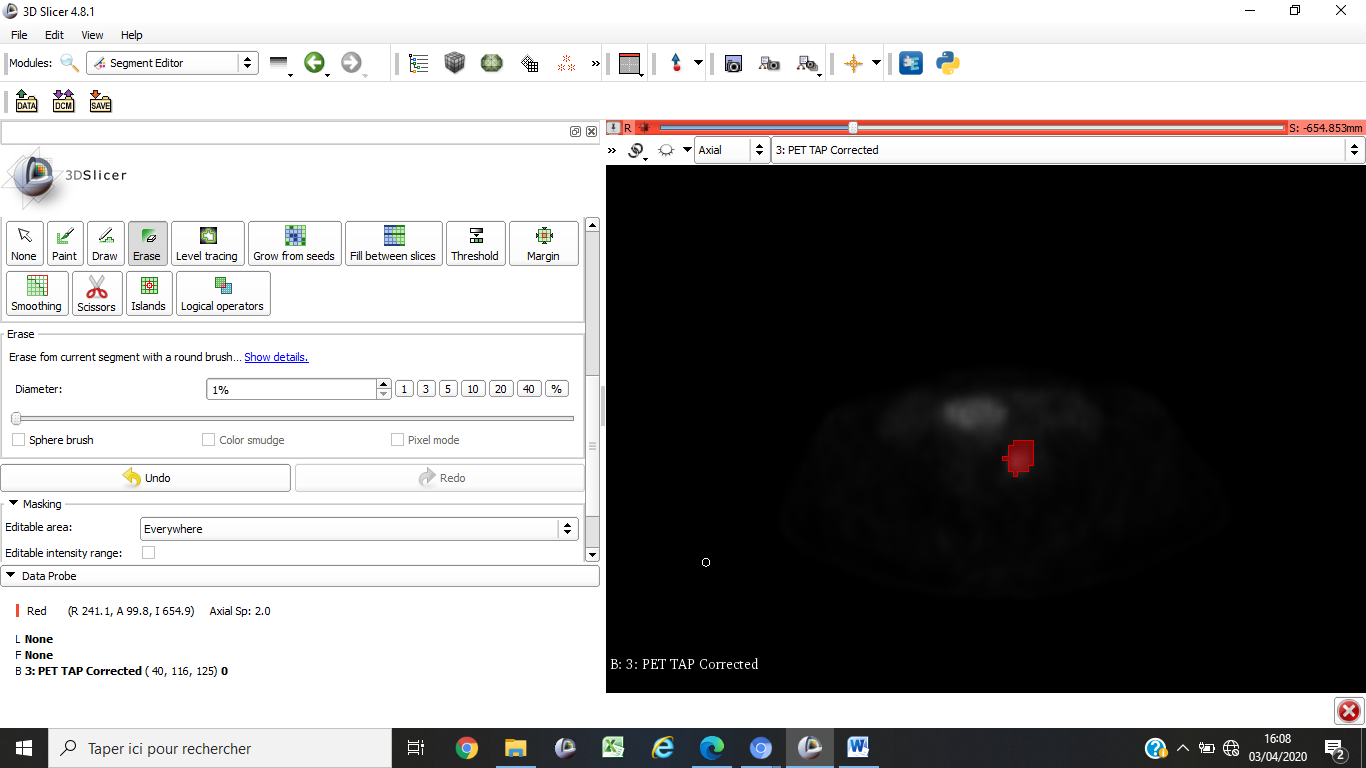


anterior

anterior


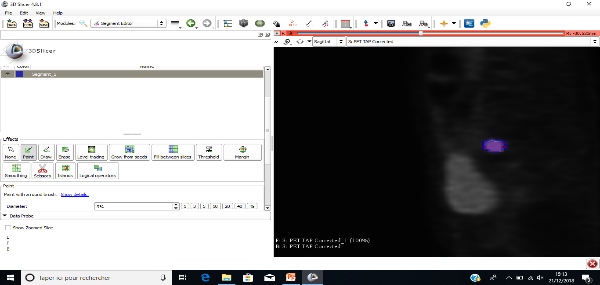

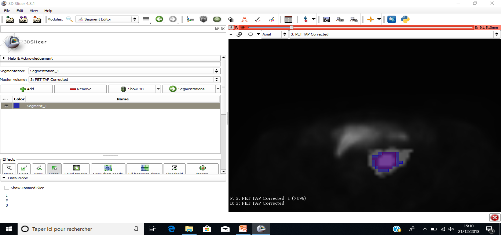


anterior

anterior

D.


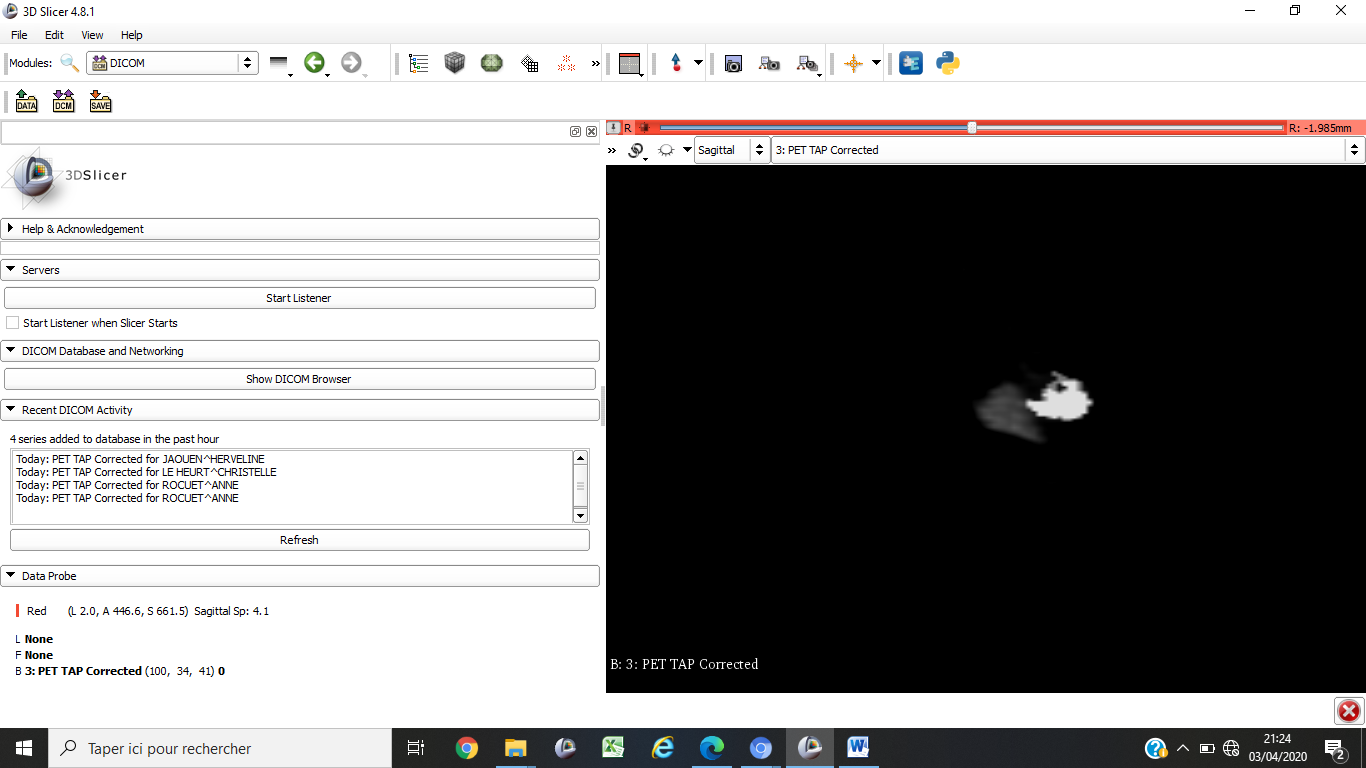

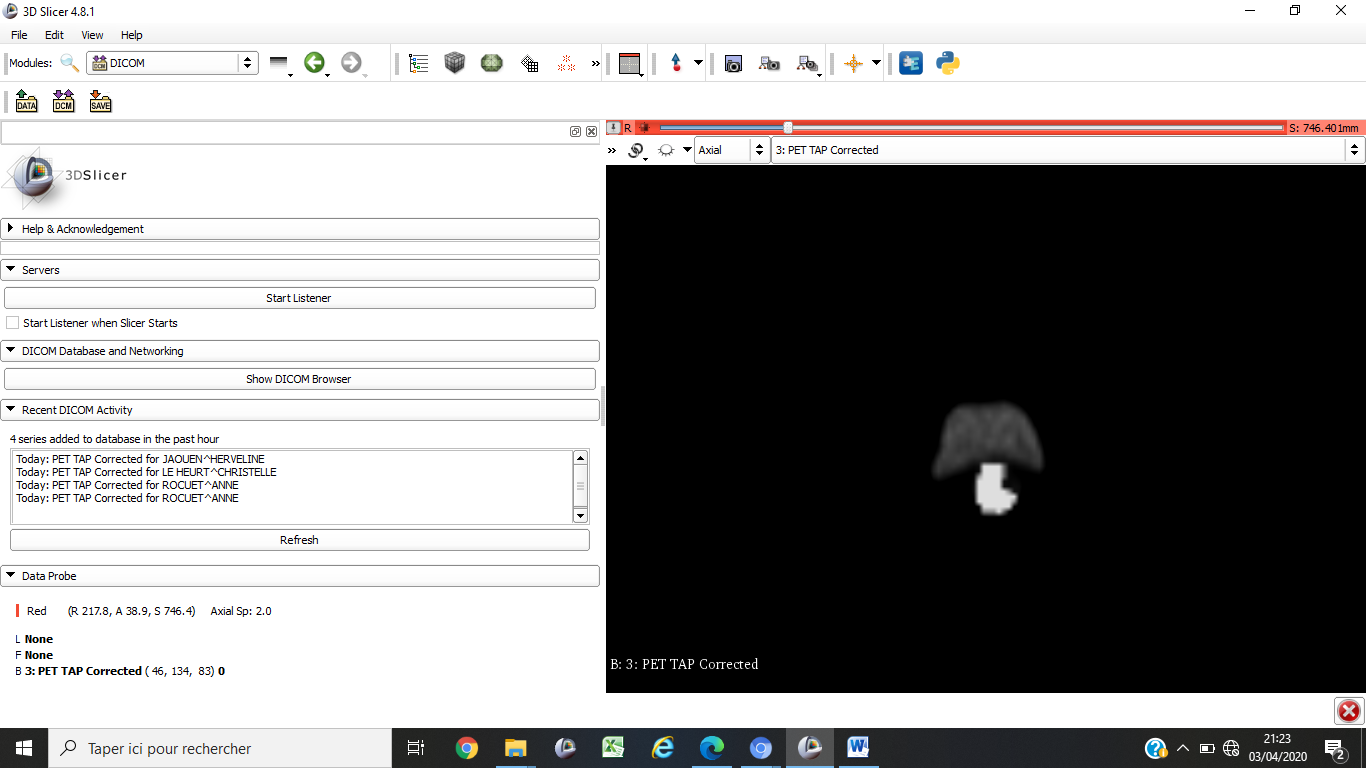


anterior

anterior


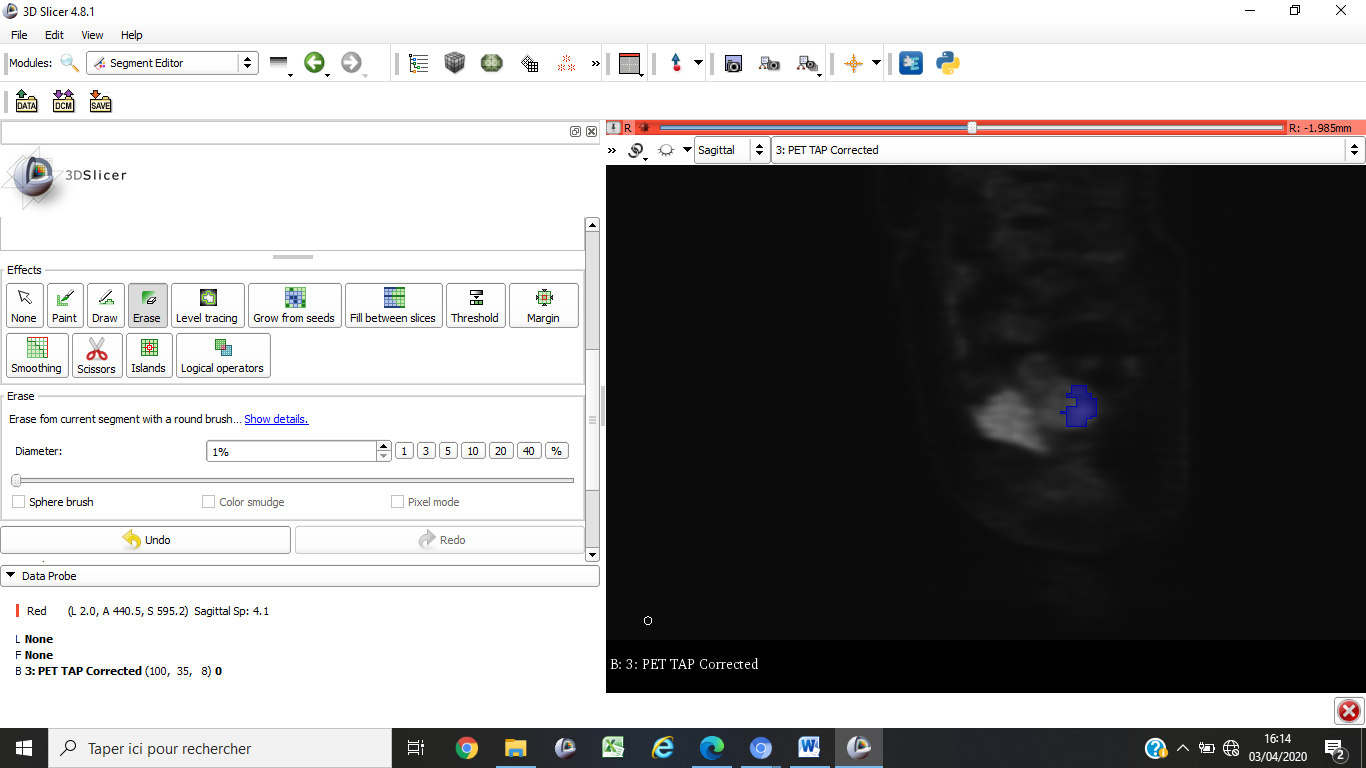

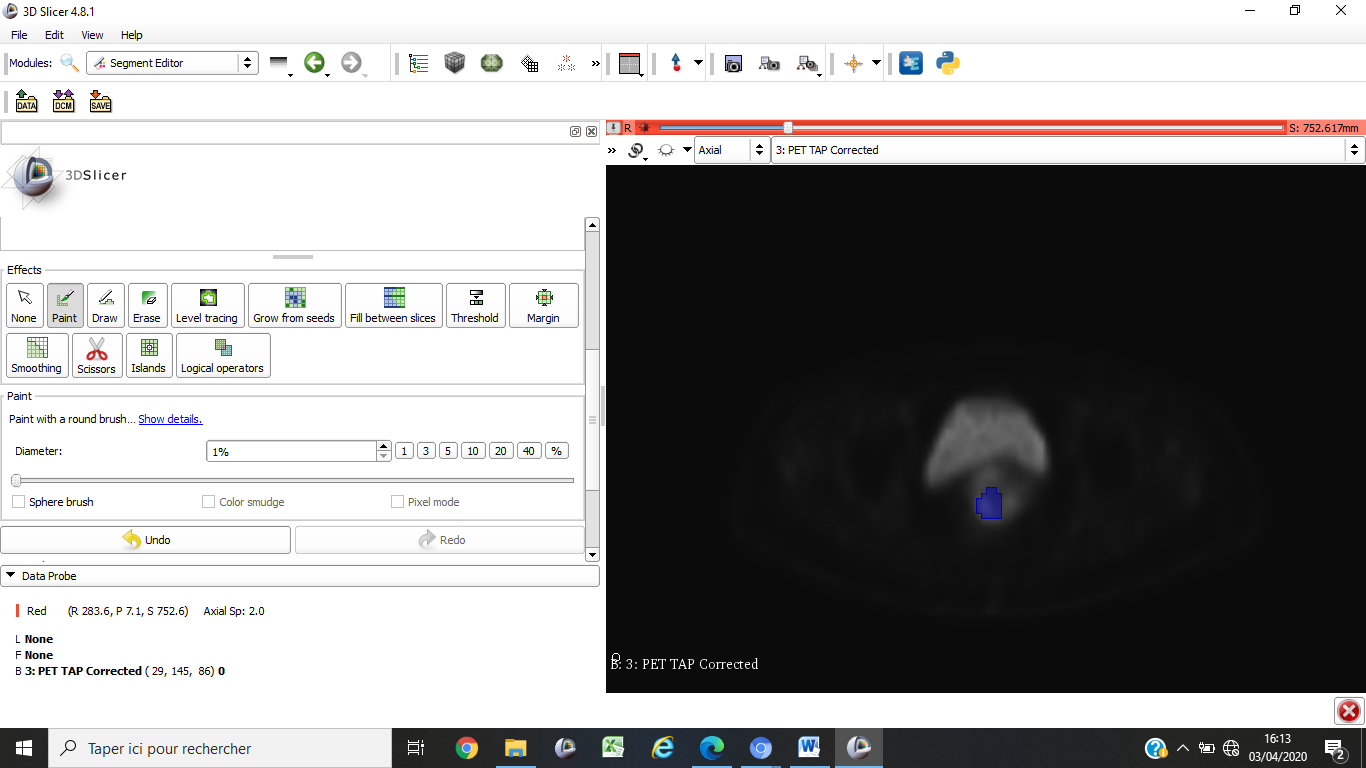


anterior

anterior


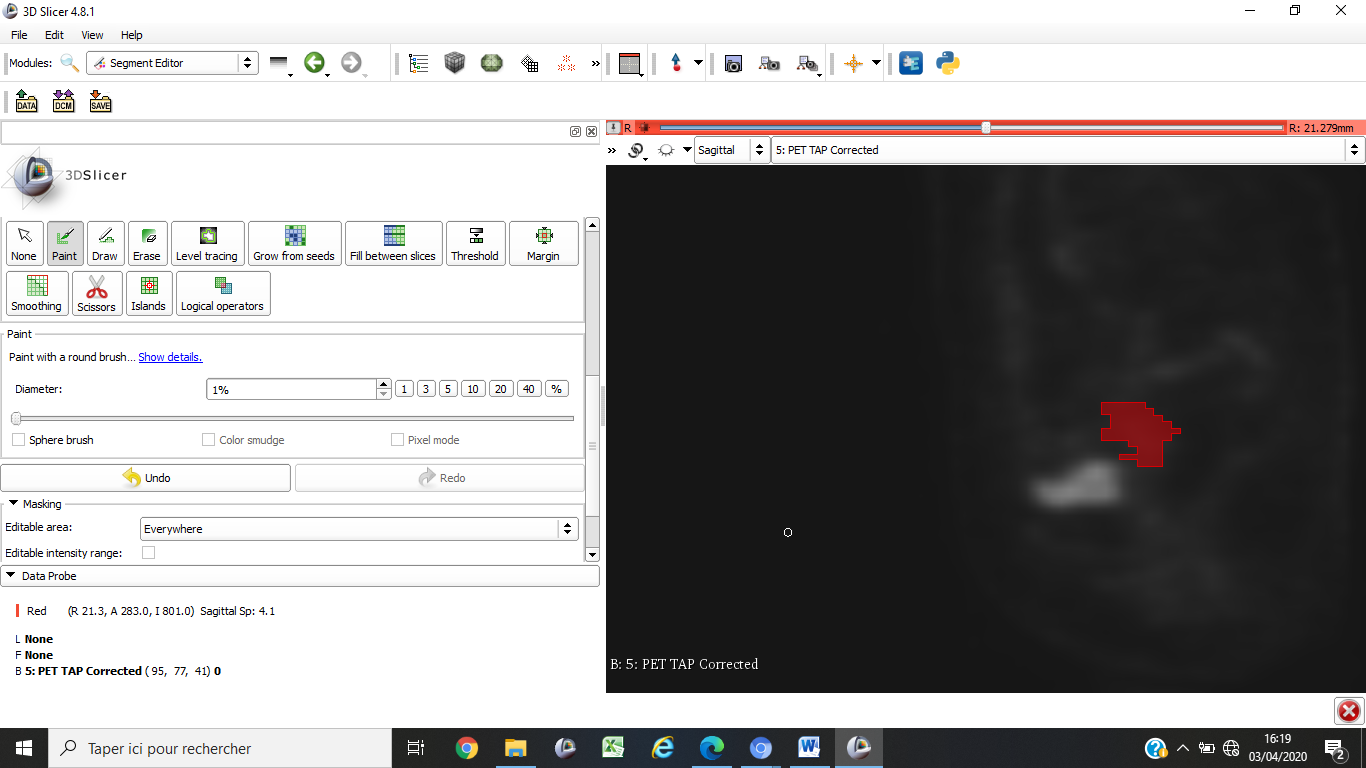

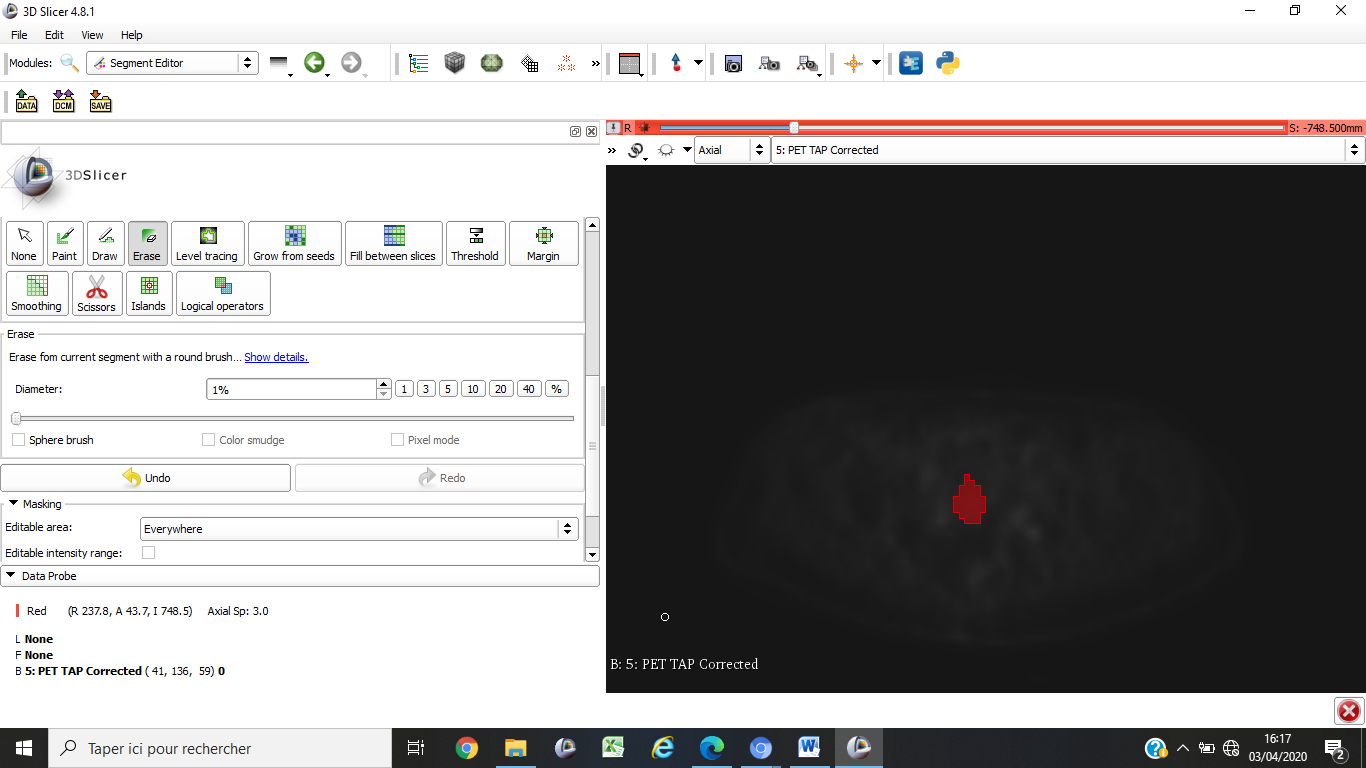


anterior

anterior


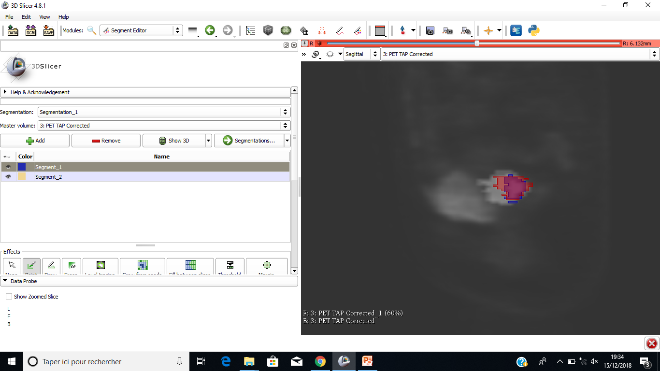

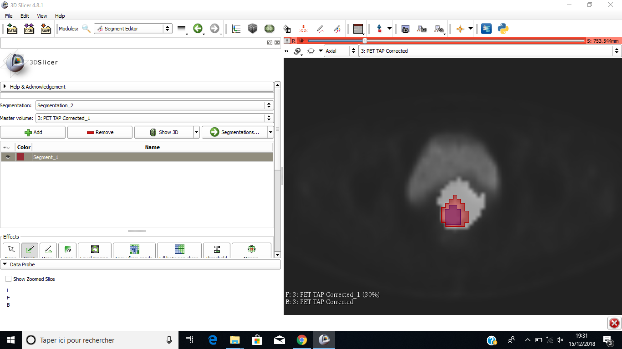


anterior

anterior

**Table S1: comparison between 2 subgroups**

|  |  | |  |  |  |  | |  | |
| --- | --- | --- | --- | --- | --- | --- | --- | --- | --- |
|  | n=14 | % | n=7 | | | | % | | *P* |
| Age median (range) | 55 (34-79) |  | 53 (32-77) | | | |  | | 0.83 |
| FIGO stage |  |  |  | | | |  | |  |
| IB1 | 1 | 7 | 0 | | | | 0 | | 0.70 |
| IB2 | 0 | 0 | 1 | | | | 14 | | 0.74 |
| IIA | 1 | 7 | 0 | | | | 0 | | 0.70 |
| IIB | 9 | 65 | 3 | | | | 44 | | 0.65 |
| IIIA | 0 | 0 | 1 | | | | 14 | | 0.74 |
| IIIB | 2 | 14 | 1 | | | | 14 | | 0.50 |
| IVA | 1 | 7 | 1 | | | | 14 | | 0.78 |
| Histology |  |  |  | | | |  | |  |
| Squamous carcinoma | 12 | 85 | 5 | | | | 71 | | 0.86 |
| Adenocarcinoma | 2 | 15 | 1 | | | | 19 | | 0.69 |
| Adenosquamous carcinoma | 0 | 0 | 0 | | | | 0 | | 1.00 |
| Clear cell carcinoma | 0 | 0 | 1 | | | | 14 | | 0.74 |
| Lymph node involvement |  |  |  | | | |  | |  |
| Uninvoled | 6 | 43 | 3 | | | | 43 | | 0.64 |
| Involved | 8 | 57 | 4 | | | | 57 | | 0.64 |
| pelvic | 6 | 75 | 2 | | | | 50 | | 0.83 |
| pelvic and para-aortic | 2 | 25 | 2 | | | | 50 | | 0.83 |
| Treatment |  |  |  | | | |  | |  |
| 3D-CRT | 10 | 71 | 5 | | | | 71 | | 0.61 |
| IMRT | 4 | 29 | 2 | | | | 29 | | 0.61 |
| EBRT dose median (range) | 45 (45-54) |  | 45 (45-54) | | | |  | | 1.00 |
| BT dose median (range) | 24 (21-26) |  | 24 (21-26) | | | |  | | 1.00 |
| Relapse |  |  |  | | | |  | |  |
| isolated local recurrence | 5 | 36 | 3 | | | | 42 | | 0.83 |
| local and nodal recurrences | 6 | 43 | 2 | | | | 29 | | 0.88 |
| local and distant recurrences | 3 | 21 | 2 | | | | 29 | | 0.89 |
| Time of relapse (months) | 6±4 |  | 6±4 | | | |  | | 1.00 |
| PET1 (mean) |  |  |  | | | |  | |  |
| SUV_max_ | 19.3±7.2 |  | 19.8±7.5 | | | |  | | 0.88 |
| SUV_mean_ | 6.5±2.8 |  | 6.7±2.9 | | | |  | | 0.88 |
| TLG | 284.4±279.5g |  | 287.3±281.8g | | | |  | | 0.98 |
| PET2 (mean) |  |  |  | | | |  | |  |
| SUV_max_ | 20.9±9.7 |  | 21.5±10.1 | | | |  | | 0.90 |
| SUV_mean_ | 8.7±3.9 |  | 9.1±4.2 | | | |  | | 0.83 |
| TLG | 184.7±277.3g |  | 186.7±279.1g | | | |  | | 0.99 |

Abbreviations: FIGO= International Federation of Gynecology and Obstetrics, 3D-RT= three-dimensional conformal radiotherapy, IMRT= intensity-modulated photon radiotherapy, EBRT= external beam radiotherapy, BT=brachytherapy, SUV_max_= maximum standardized uptake value, SUV_mean_= mean standardized uptake value, TLG=Total Lesion Glycolysis
